# Addiction to DUSP1 protects JAK2V617F-driven polycythemia vera progenitors against inflammatory stress and DNA damage, allowing chronic proliferation

**DOI:** 10.1038/s41388-019-0813-7

**Published:** 2019-04-09

**Authors:** J. Stetka, P. Vyhlidalova, L. Lanikova, P. Koralkova, J. Gursky, A. Hlusi, P. Flodr, S. Hubackova, J. Bartek, Z. Hodny, V. Divoky

**Affiliations:** 1 Department of Biology, Faculty of Medicine and Dentistry, Palacky University, Olomouc, Czech Republic; 2 Department of Histology and Embryology, Faculty of Medicine and Dentistry, Palacky University, Olomouc, Czech Republic; 3 Laboratory of Cell and Developmental Biology, Institute of Molecular Genetics of the ASCR, v. v. i., Prague, Czech Republic; 4 Institute of Molecular and Translational Medicine, Faculty of Medicine and Dentistry, Palacky University, Olomouc, Czech Republic; 5 Department of Hemato-Oncology, University Hospital and Faculty of Medicine and Dentistry, Palacky University, Olomouc, Czech Republic; 6 Department of Clinical and Molecular Pathology, University Hospital and Faculty of Medicine and Dentistry, Palacky University, Olomouc, Czech Republic; 7 Laboratory of Molecular Therapy, Institute of Biotechnology, BIOCEV, Czech Academy of Sciences, Prague-West, 252 50 Czech Republic; 8 Danish Cancer Society Research Center, DK-2100 Copenhagen, Denmark; 9 Laboratory of Genome Integrity, Institute of Molecular Genetics of the ASCR, v. v. i., Prague, Czech Republic; 10 Division of Genome Biology, Department of Biochemistry and Biophysics, Science for Life Laboratory, Karolinska Institute, Stockholm, Sweden

**Keywords:** Cancer microenvironment, Inflammation, DNA damage response, Myeloproliferative disease

## Abstract

Inflammatory and oncogenic signaling converge in disease evolution of BCR–ABL-negative myeloproliferative neoplasms, clonal hematopoietic stem cell disorders characterized by gain-of-function mutation in *JAK2* kinase (JAK2V617F), with highest prevalence in patients with polycythemia vera (PV). Despite the high risk, DNA-damaging inflammatory microenvironment, PV progenitors tend to preserve their genomic stability over decades until their progression to post-PV myelofibrosis/acute myeloid leukemia. Using induced pluripotent stem cells-derived CD34^+^ progenitor-enriched cultures from JAK2V617F^+^ PV patient and from JAK2 wild-type healthy control, CRISPR-modified HEL cells and patients’ bone marrow sections from different disease stages, we demonstrate that JAK2V617F induces an intrinsic IFNγ- and NF-κB-associated inflammatory program, while suppressing inflammation-evoked DNA damage both in vitro and in vivo. We show that cells with JAK2V617F tightly regulate levels of inflammatory cytokines-induced reactive oxygen species, do not fully activate the ATM/p53/p21waf1 checkpoint and p38/JNK MAPK stress pathway signaling when exposed to inflammatory cytokines, suppress DNA single-strand break repair genes’ expression yet overexpress the dual-specificity phosphatase (DUSP) 1. RNAi-mediated knock-down and pharmacological inhibition of DUSP1, involved in p38/JNK deactivation, in HEL cells reveals growth addiction to DUSP1, consistent with enhanced DNA damage response and apoptosis in DUSP1-inhibited parental JAK2V617F^+^ cells, but not in CRISPR-modified JAK2 wild-type cells. Our results indicate that the JAK2V617F^+^ PV progenitors utilize DUSP1 activity as a protection mechanism against DNA damage accumulation, promoting their proliferation and survival in the inflammatory microenvironment, identifying DUSP1 as a potential therapeutic target in PV.

## Introduction

Inflammatory signaling is a common mechanism fueling genotoxic stress and contributing to tumorigenesis including transformation in the hematopoietic system [1]. Three subtypes of myeloproliferative neoplasms (MPNs): polycythemia vera (PV), essential thrombocythemia (ET), and primary myelofibrosis (PMF), are clonal hematological disorders with overlapping phenotypes characterized by oncogenic Janus kinase (JAK)-signal transducers and activators of transcription (STAT)-mediated signaling, associated chronic inflammation, long clinical course, and long-term disease evolution [2]. The most frequent MPN driving mutation, the gain-of-function JAK2V167F, activates type-1 myeloid cytokine receptors and is detected in >95% of PV and 50–60% ET and PMF as an early somatic event [3]. Dysregulation of this key cytokine receptor signaling fuels formation of a bone marrow (BM) microenvironment with aberrant synthesis of inflammatory cytokines and chemokines, triggering persistent, systemic inflammatory response marked by elevated inflammatory markers in circulation [4, 5]. In a mouse model, inflammatory factors contribute to remodeling of the BM microenvironment into leukemic niche that inhibits normal hematopoiesis and favors leukemic stem cell formation and myelofibrosis [6, 7]. However, despite the pronounced chronic inflammation, the two “proliferative” MPN disease types, PV and ET, with overproduction of mature, functional myeloid elements, have a relatively low cumulative incidence of blast transformation and fibrotic progression [8]. We therefore hypothesized that the JAK2V617F-driven activation of cell autonomous and non-cell autonomous inflammatory programs induces some feedforward protective loop that guards PV progenitors from cell-intrinsic and cell-extrinsic DNA-damaging stimuli, providing a barrier that prevents cell cycle arrest, delays stress-induced regulatory hematopoietic circuits associated with myelofibrosis [9, 10], and decreases DNA damage accumulation associated with rapid malignant transformation [11].

Human induced pluripotent stem cells (iPSCs) have been widely used for disease modeling, including PV [12], and transcriptional profiling, cell cycle, and DNA damage response (DDR) studies in specialized cell types differentiated from iPSCs [13]. Here, we compared differentiated, PV patient-specific iPSCs with a homozygous JAK2V617F constitution and gender-matched iPSCs with wild-type JAK2 (JAK2wt), along with genetically modified JAK2V617F-expressing HEL cells and immunohistochemical (IHC) staining of PV patients’ BM, to investigate the hierarchy and consequences of the activated inflammatory signature. We found an increased reactive oxygen species (ROS)-buffering capacity and overexpression of the dual-specificity phosphatase (DUSP) 1 in JAK2V617F cells as candidate mechanisms keeping fast-cycling PV progenitors in a proliferative phase despite the surrounding genotoxic inflammatory microenvironment.

## Results

### Validation of the iPSC-derived hematopoietic differentiation model

PV patient-specific JAK2V617F^+^ (PVB1.4) and control JAK2wt (BXS0116) iPSC lines, reprogrammed from BM CD34^+^ cells, were differentiated for 9 days (hereafter d9) into CD34^+^ hematopoietic progenitors (Supplementary Materials and Methods) [14]. Contaminating cell types from other than hematopoietic lineages were present, however, our protocol generated cultures with comparable abundance of CD34^+^ hematopoietic progenitors in both JAK2wt and V617F^+^-differentiated cultures (hereafter referred to as CD34^+^ progenitor-enriched cultures, P-ECs), with comparable abundance of other key hematopoietic surface antigens and transcripts for early hematopoietic markers and signaling pathways regulating stem cell pluripotency (Supplementary Fig. 1a–d). As expected, contrary to JAK2wt cells, the JAK2V617F^+^ CD34^+^ P-ECs at d9 exhibited markers of myeloid/erythroid lineage biased differentiation (Supplementary Fig. 1e). Furthermore, both PVB1.4 and BXS0116 iPSC lines showed unimpaired activation of DDR markers in both JAK2wt and JAK2V617F^+^ undifferentiated iPSC clones following DNA-damaging X-ray irradiation (Supplementary Fig. 1f), suggesting functionality of DDR signaling in our model. Next, we treated the model cell lines with interferon-γ (IFNγ), tumor necrosis factor-alpha (TNFα), and transforming growth factor-beta 1 (TGFβ1), the upstream principal regulators of the inflammatory program and microenvironment alterations in MPN [15–17]. IFNγ, TNFα, and TGFβ1 are known to induce genotoxic stress, DDR signaling and senescence in both normal and tumor cells [18] and we hypothesized that the JAK2V617F^+^ P-ECs are refractory to such treatment.

### JAK2V617F^+^ progenitors activate an inflammatory signature intrinsically by wake of IFNγ transcription and IFNγ-dependent STAT1 signaling

**Fig. 1:**
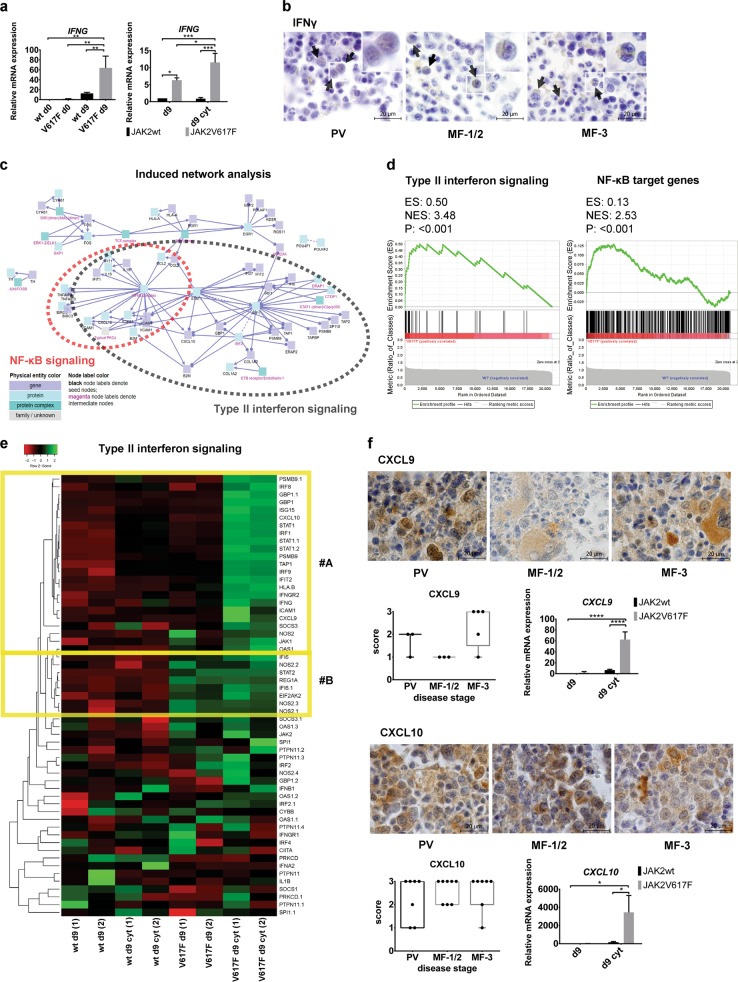
Intrinsic and proinflammatory cytokine-induced expression of IFNγ and IFNγ-regulatory program in JAK2V617F^+^ progenitors. **a** Left bar graph: intrinsic *IFNG* mRNA expression (mean ± SD; *n* = 3 independent experiments) in d9 JAK2V617F^+^ CD34^+^ P-ECs compared with d9 JAK2wt CD34^+^ P-ECs and with undifferentiated JAK2wt and V617F^+^ iPSC lines (d0). Right bar graph: *IFNG* mRNA expression in three independent experiments after treatment with IFNγ, TNFα, and TGFβ1 inflammatory cytokines (d9 cyt). *P ≤ 0.05, **P ≤ 0.01, ***P ≤ 0.001, one-way and two-way ANOVA, respectively. **b** Representative IHC staining of BM sections for IFNγ among different disease stages of PV progression. Scale bar, 20 µm. Insets in each panel show magnification of individual IFNγ-producing cells. **c** Visualization of induced network module analysis made by ConsensusPathDB using differentially upregulated genes (logFC > 1, *q* < 0.1 cut-off) between JAK2wt and V617F^+^ CD34^+^ P-ECs treated with inflammatory cytokines (d9 cyt) as seed genes with seed nodes (black) and intermediate nodes (magenta) connected through functional and physical links. Red circle depicts visualized nodes associated with NF-κB pathway, with significant type II interferon signaling genes overlap (gray circle). **d** Left: GSEA plot of type II interferon signaling gene set members (WikiPathway, *n* = 37) in d9 cyt JAK2V617F^+^ and JAK2wt CD34^+^ P-ECs. Right: GSEA of d9 cyt JAK2V617F^+^ and JAK2wt CD34^+^ P-ECs using a set of 424 NF-κB target genes (http://www.bu.edu/nf-kb/gene-resources/target-genes/). **e** Heatmap representation of differential expression of type II interferon signaling gene set members in d9 JAK2wt and JAK2V617F^+^ CD34^+^ P-ECs, untreated (d9) or treated with inflammatory cytokines (d9 cyt). Columns in the heatmap represent individual samples in experimental duplicates (1, 2). **f** IHC staining of CXCL9 and CXCL10 in representative BM sections from patients along the progression of PV. Scale bar, 20 µm. Boxplots show quantification of the number of cells expressing CXCL9 and CXCL10 in sections of patients from grouped disease stages. The bar graphs show mRNA expression (mean ± SD) of three independent experiments for *CXCL9* and *CXCL10* in d9 cyt JAK2V617F^+^ CD34^+^ P-ECs, compared with untreated (d9) or treated (d9 cyt) JAK2wt CD34^+^ P-ECs. *P ≤ 0.05, ****P ≤ 0.0001, Mann–Whitney test (IHC) and two-way ANOVA (qRT-PCR). See also Supplementary Fig. 1

Subpopulations of hematopoietic stem cells (HSCs) and progenitors exhibit high IFN responsiveness maintained by intrinsic expression of a subset of IFN-stimulated genes [19, 20]. As the JAK2V617F^+^ cells display high STAT1 activation [15, 21] and the most highly, cell-autonomously induced cytokine in JAK2V617F^+^ progenitors is CXCL10 [22, 23], an IFNγ-inducible chemokine [24], we first tested intrinsic and inflammatory cytokine-inducible expression of IFNγ in JAK2V617F^+^ progenitors. D9 derivatives of the JAK2V617F^+^ cultures intrinsically expressed *IFNG*, which was further increased after treatment with inflammatory cytokines (IFNγ, TNFα, and TGFβ1 treatment of d9 CD34^+^ P-ECs for 24 h, hereafter d9 cyt) specifically in the JAK2V617F^+^ CD34^+^ P-ECs but not in their JAK2wt counterparts (Fig. 1a). IFNγ-producing immature myeloid cells were present in BM at all PV disease stages (PV, MF-1/2 PV with fibrosis grade 1 and 2, MF-3 PV with fibrosis grade 3/post-PV MF) [25] (Fig. 1b). IFNγ staining also colocalized with the CD34 antigen in embryoid bodies (EBs) derived from the JAK2V617F^+^ iPSCs (Supplementary Fig. 1g). STAT1 phosphorylation on tyrosine 701 was induced by IFNγ alone or combined with TGFβ1 and/or TNFα more robustly in the JAK2V617F^+^ CD34^+^ P-ECs than in the JAK2wt cells (Supplementary Fig. 1h).

To gain more insights into the expression program differentially regulated between d9 JAK2wt and JAK2V617F^+^ CD34^+^ P-ECs, in medium without or with inflammatory cytokines, we performed whole-genome transcriptional profiling. Top 20 differentially upregulated genes included overexpression of type II (IFNγ)-stimulated genes in the JAK2V617F^+^ versus the JAK2wt P-ECs (Supplementary Fig. 1i). Among the affected networks identified using the ConsensusPathDB pathway analysis, IFNγ and nuclear factor-kappa B (NF-κB) signaling pathways were enriched for differentially overexpressed genes in d9 cyt JAK2V617F^+^ CD34^+^ P-ECs as compared with JAK2wt d9 cyt cells (Fig. 1c and Supplementary Fig. 1j). IFNγ and NF-κB signaling gene set members were significantly upregulated in d9 cyt JAK2V617F^+^ compared with JAK2wt CD34^+^ P-ECs (determined by gene set enrichment analysis (GSEA), Fig. 1d). An expression heatmap for IFNγ signaling genes showed two clusters: cluster #A with genes exclusively induced in the JAK2V617F^+^ cells upon treatment with inflammatory cytokines, and cluster #B containing intrinsically upregulated IFNγ-dependent genes in the JAK2V617F^+^ cells (Fig. 1e). These experiments suggested cell-intrinsic IFNγ priming driven by JAK2V617F.

### Cooperation of IFNγ, TNFα and/or TGFβ1 triggers robust expression of pro-fibrogenic chemokines in JAK2V617F^+^ CD34^+^ P-ECs

Among the most differentially upregulated NF-κB target genes (Supplementary Fig. 1k) in the d9 cyt JAK2V617F^+^ CD34^+^ P-ECs, which overlapped with the induced IFNγ signaling gene set members were pro-fibrogenic chemokines CXCL9 and CXCL10. Both chemokines were highly expressed in PV patients’ samples; the level of CXCL9 was highest in progressed post-PV MF-3 while CXCL10 was expressed constitutively across disease stages (Fig. 1f). We tested whether overexpression of these chemokines in the JAK2V617F^+^ P-ECs is mainly due to IFNγ responsiveness (or other individual cytokines) of cells exhibiting an intrinsic IFNγ priming state or it reflects a cooperation between IFNγ and upstream pro-fibrogenic regulators TNFα and TGFβ1 [24, 26–28]. In vivo, consistently with earlier studies [29, 30], we detected progressively increased expression of TNFα with staining restricted to fibrotic tissue and constitutive expression of TGFβ1 during the disease progression (Supplementary Fig. 1l). While IFNγ, TNFα, or TGFβ1 used alone did not elicit any significant pro-fibrogenic response (data not shown), combinations of IFNγ with TNFα and IFNγ with TNFα and TGFβ1 robustly induced *CXCL9* and *CXCL10* in the JAK2V617F^+^ progenitors (Fig. 1f). As IL6 and CCL3 were also shown to be an important part of the proinflammatory profile in patients with MPN [5], we have analyzed their expression in BM sections from patients at different PV disease stages. Both cytokines were constitutively present across the disease stages with high expression of CCL3 in basophil-like progenitor cells, as previously described in CML [31], and modest expression of IL6 (Supplementary Fig. 1l).

These results supported a powerful pro-fibrogenic response of the JAK2V617F^+^ progenitors and a cooperation of inflammatory mediators in disease evolution, including the role of CXCL10 and CXCL9 in fibrogenesis.

### JAK2V617F-mediated protection against inflammation-evoked DNA damage accumulation and the DDR

**Fig. 2:**
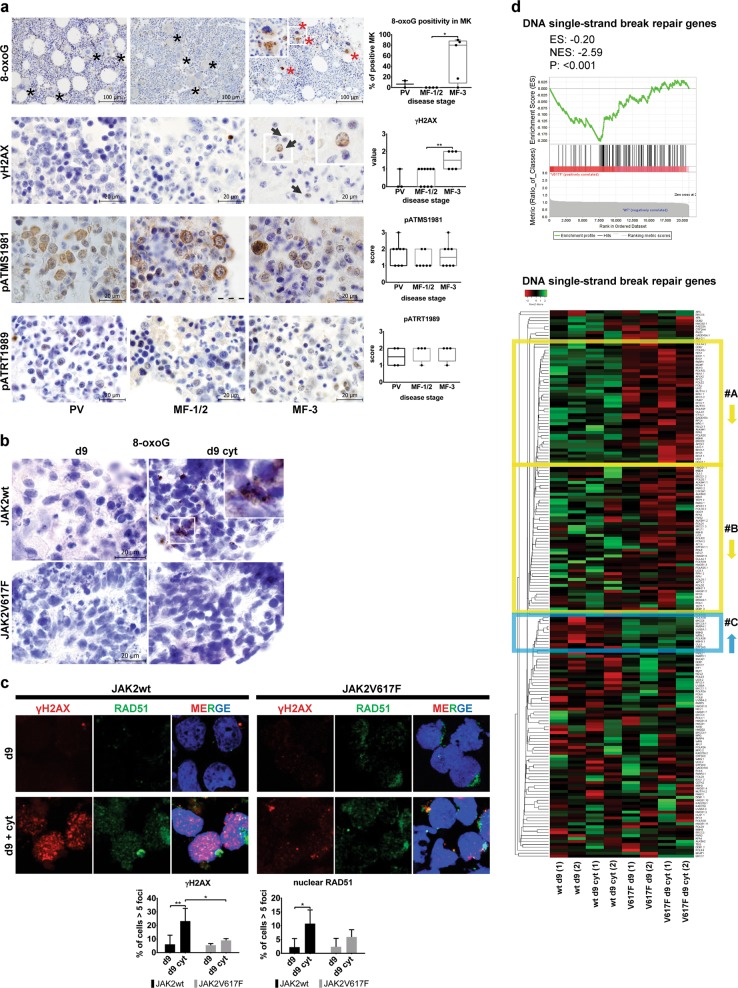
Protection against inflammation-induced DNA damage accumulation and DDR in PV cells. **a** Immunohistochemistry staining for oxidative DNA damage and DDR markers in PV, MF-1/2, and post-PV MF-3 patients. In 8-oxoguanine (8-oxoG) staining, red asterisks mark 8-oxoG-positive megakaryocytes (MKs), black asterisks denote negative MKs; scale bar, 100 µm. In γH2AX staining, inset in MF-3 panel shows magnification of cells stained positive for γH2AX foci; scale bar, 20 µm. pATMS1981 staining revealed cytoplasmic and pATRT1989 nuclear positivity; scale bars, 20 µm. Boxplots show quantification of the number of cells expressing indicated markers in sections of patients from grouped disease stage*s.* *P ≤ 0.05, **P ≤ 0.01, Mann–Whitney test. **b** Immunostaining for 8-oxoG of paraffin-embedded EBs derived from d9 and d9 cyt JAK2wt and V617F^+^ iPSCs. **c** γH2AX (red) and RAD51 (green) staining of d9 and d9 cyt JAK2wt and JAK2V617F^+^ CD34^+^ P-ECs. Charts show percentage of cells ± SD in three independent experiments with more than five γH2AX or RAD51 foci per cell in nucleus. *P ≤ 0.05, **P ≤ 0.01, two-way ANOVA. **d** Top: GSEA of d9 cyt JAK2V617F^+^ and JAK2wt CD34^+^ P-ECs using a set of 112 single-strand repair genes [37]. Bottom: Heatmap representation of single-strand repair gene set expression in d9 and d9 cyt JAK2wt and JAK2V617F^+^ CD34^+^ P-ECs. Columns represent individual samples in experimental duplicates (1, 2). Two large differentially downregulated clusters of genes in V617F^+^ samples are highlighted by yellow box with low variance between experimental duplicates (#A) and higher variance (#B). Blue box delimits cluster of genes upregulated with low variance between experimental duplicates in V617F^+^ samples (#C). See also Supplementary Fig. 2

Although some studies reported abundant JAK2V617F-dependent oxidative DNA lesions due to enhanced ROS generation [32] and increased homologous recombination (HR) activity and genetic instability fueled by the oncogenic JAK2V617F in MPN [33], others have questioned such features of the JAK2V617F-expressing progenitors [34]. To establish whether and how JAK2V617F causes oncogenic stress and influences sensitivity or resistance to inflammation-evoked DNA damage, we first analyzed the overall degree of DDR activation in BM sections from patients at different PV disease stages. Oxidative DNA damage (8-oxoguanine, 8-oxoG) was barely detectable in PV and MF-1/2, and was only increased in post-PV MF-3, due to positivity of megakaryocytes (Fig. 2a and Supplementary Fig. 2a). The global DDR marker, Ser 139-phosphorylated histone H2AX (γH2AX), showed similar patterns, with moderate positive staining only in post-PV MF-3 samples (Fig. 2a). The activated form of ATM, Ser 1981-phosphorylated ATM (pATMS1981), was constitutively present in cytoplasm in all disease stages, consistent with ROS-mediated activation [35], whereas the activated form of ATR, Thr 1989-phosphorylated ATR (pATRT1989), showed low constitutive nuclear staining (Fig. 2a). These data suggested a mild degree of oxidative and replication stress, not converted into double-strand DNA breaks (absence of γH2AX foci) in PV and MF-1/2 (consistent with ongoing proliferation), and activated DDR signaling only at the post-PV MF-3 disease state.

Next, we determined oxidative damage and DDR activation in the differentiated JAK2wt and JAK2V617F^+^ iPSCs exposed to inflammatory cytokines. 8-oxoG staining was high in EBs derived from the JAK2wt iPSCs but undetectable in the V617F^+^ counterparts (Fig. 2b). Assessment of DDR in vitro confirmed lower DNA damage (fewer γH2AX foci) in the d9 cyt JAK2V617F^+^ CD34^+^ P-ECs, compared with JAK2wt CD34^+^ P-ECs (Fig. 2c and Supplementary Fig. 2b), whereas inducing bystander DNA damage to the JAK2wt CD34^+^ P-ECs upon co-culture (Supplementary Fig. 2c). RAD51 recruitment to DNA damage sites, marked by γH2AX foci as readout for HR efficiency upon sequential staining [36], was not significantly altered (Fig. 2c and Supplementary Fig. 2b). With RAD51 accumulation following the trends of γH2AX levels these data suggest intact regulation of HR in the JAK2V617F^+^ cells.

To examine whether observed protection against inflammation-evoked DNA damage in the JAK2V617F^+^ cells is reflected in the global expression of DDR and DNA repair genes, we evaluated transcripts of 369 genes distributed to categories as described [37]. In individual heatmaps, clusters of upregulated genes in the d9 cyt JAK2wt CD34^+^ P-ECs were larger than in the JAK2V617F^+^ CD34^+^ P-ECs (Fig. 2d and Supplementary Fig. 2d). DNA single-strand repair represented the most differentially expressed gene (DEG) set, as GSEA revealed enrichment for downregulated genes in the JAK2V617F^+^ CD34^+^ P-ECs (Fig. 2d).

We tested whether apparent DNA damage protection of V617F^+^ cells is efficient only for inflammation-induced threshold dose of DNA damage, and thus represents adaptations to cell autonomous and microenvironment-dependent inflammatory stress in PV. Indeed, X-ray irradiation of the d9 JAK2V617F^+^ CD34^+^ P-ECs induced DDR signaling comparable or higher than in the JAK2wt cells (Supplementary Fig. 2e, f), suggesting that suprathreshold DNA damage activates robust DDR in JAK2V617F^+^ cells.

### Inflammatory cytokines inhibit cell cycle of JAK2wt CD34^+^ P-EC cells but fail to activate the p53/p21waf1 checkpoint in the JAK2V617F^+^ cells

**Fig. 3:**
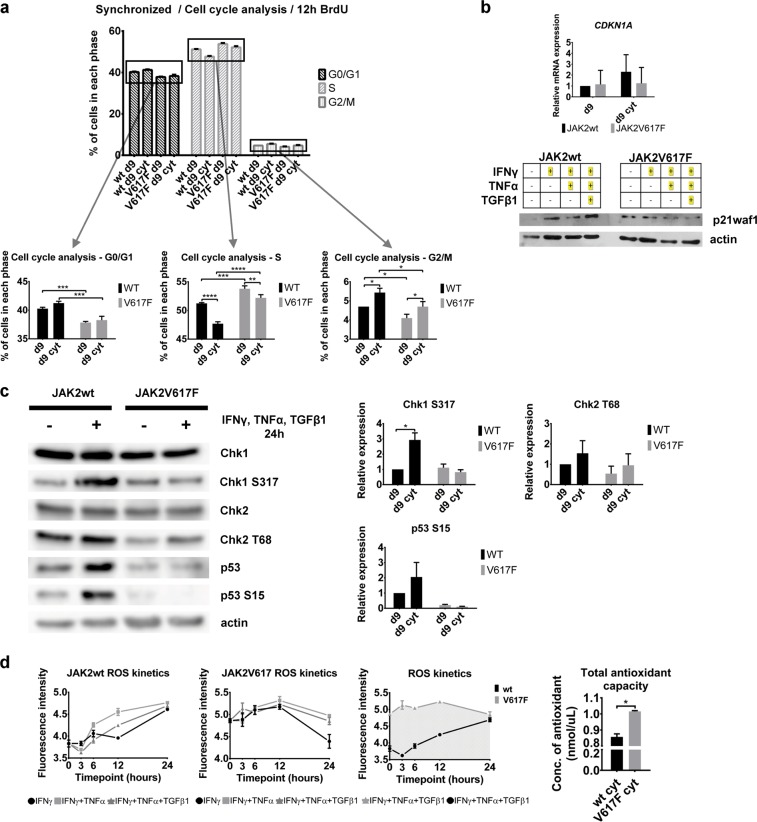
Characterization of cell cycle progression, checkpoint activation and ROS production and buffering in JAK2wt and JAK2V617F^+^ CD34^+^ P-ECs. **a** Changes of cell cycle profiles of synchronized CD34^+^ P-EC cells untreated (d9) or treated with IFNγ, TNFα and TGFβ1 for 24 h (d9 cyt). Bar charts show percentage of cell numbers ± SD from three independent experiments in each phase of cell cycle. To further express the differences in each phase of cell cycle, the top of the chart bars were zoomed-in on separate charts. *P ≤ 0.05, **P ≤ 0.01, ***P ≤ 0.001, ****P ≤ 0.0001, two-way ANOVA. **b** Top: Quantitative RT-PCR analysis of *CDKN1A* (p21waf1) gene expression in day d9 or d9 cyt JAK2wt and JAK2V617F^+^ CD34^+^ P-ECs. Values are shown as a mean ± SD of three independent experiments. Bottom: Representative immunoblotting analysis with protein levels of p21waf1 in JAK2wt and JAK2V617F^+^ CD34^+^ P-ECs upon treatment with IFNγ, TNFα, and/or TGFβ1 in various combinations. **c** Western blotting analysis of total and phosphorylated forms of checkpoint proteins (Chk1, Chk2, and p53) in d9 and d9 cyt JAK2wt and JAK2V617F^+^ CD34^+^ P-ECs. Charts show Chk1 S317, Chk2 T68, and p53 S15 relative expression (mean ± SD, *n* = 2 independent experiments) normalized to actin. *P ≤ 0.05, two-way ANOVA. **d** Left: Kinetics of ROS production in three independent experiments presented as a mean ± SD of samples’ fluorescence intensities in JAK2wt and JAK2V617F^+^ CD34^+^ P-ECs treated with defined combinations of inflammatory cytokines for 3, 6, 12, and 24 h (left two charts); comparison of different kinetics of ROS production in JAK2wt and JAK2V617F^+^ CD34^+^ P-ECs after treatment with IFNγ, TNFα, and TGFβ1 for 24 h (right chart). Right: Total antioxidant capacity (concentration of antioxidant ± SD, *n* = 2 independent experiments) in JAK2wt and JAK2V617F^+^ CD34^+^ P-ECs treated with inflammatory cytokines for 24 h. *P *≤* 0.05, unpaired Student’s *t-*test (two-tailed). See also Supplementary Fig. 3

The observed absence of global DDR markers (including the lack of nuclear pATMS1981) in PV progenitors suggested unimpaired cell cycle progression of the JAK2V617F^+^ cells rather than robust cell cycle interference, which would otherwise be expected to occur in an inflammatory environment [18, 38]. To analyze cell cycle progression of d9 and d9 cyt JAK2wt or JAK2V617F^+^ CD34^+^ P-EC cells, we synchronized the cells using growth factor withdrawal. To release from arrest, medium with growth factors and 5-bromo-2′-deoxyuridine (BrdU) was added and cell cycle progression monitored by flow cytometry (see Supplementary Fig. 3a for schematics of the experimental setup). The JAK2V617F^+^ CD34^+^ P-EC cells revealed prolonged S-phase duration (Fig. 3a), consistent with an earlier study [39]. In contrast to the JAK2wt CD34^+^ P-EC cells, the mutant d9 cyt cells lack activation of the p53/p21waf1 checkpoint (Fig. 3b, c). Low, but constitutive activation of Ser 317-phosphorylated Chk1 (pChk1S317), an ATR target site [40] is consistent with the observed constitutive pATRT1989 nuclear staining (Fig. 2a), marking proliferating cells enriched in S phase.

These data suggested that in the JAK2V617F^+^ cells inflammatory factors induce only modest degree of DDR signaling, compatible with ongoing proliferation, thereby preventing (in the proliferative disease state) the buildup of more robust, ATM/p53/p21waf1-mediated signaling that could trigger cell cycle arrest and senescence.

### The JAK2V617F^+^ CD34^+^ P-ECs tightly regulate elevated ROS

Inflammation associated with the JAK2V617F^+^ status was reported to increase ROS accumulation in the hematopoietic compartment [32]. We hypothesized that the JAK2V617F^+^ progenitors must actively upregulate antioxidant systems to avoid detrimental effects of oxidative stress, even more than in other inflammation-associated cancers [41]. Kinetic measurements of ROS levels in the d9 cyt JAK2wt and JAK2V617F^+^ CD34^+^ P-ECs revealed that while ROS levels increase over time of cytokine exposure in the JAK2wt cells, the JAK2V617F^+^ cells maintained a relatively high yet stable ROS levels (Fig. 3d). This was associated with a significantly increased antioxidant capacity of the JAK2V617F^+^ CD34^+^ P-ECs when compared with the JAK2wt controls (Fig. 3d). Accordingly, we observed a differentially upregulated expression of peroxiredoxins, cyclooxygenases, thioredoxins, and catalase in the JAK2V617F^+^ CD34^+^ P-ECs. Further analysis of antioxidant defense enzymes revealed several enzyme activities upregulated in the JAK2V617F^+^ CD34^+^ P-ECs (Supplementary Fig. 3b).

These results suggested relatively increased but tightly controlled ROS levels in the JAK2V617F^+^ compartment.

### DUSP1 protects the JAK2V617F^+^ progenitors against stress response signaling and DDR

**Fig. 4:**
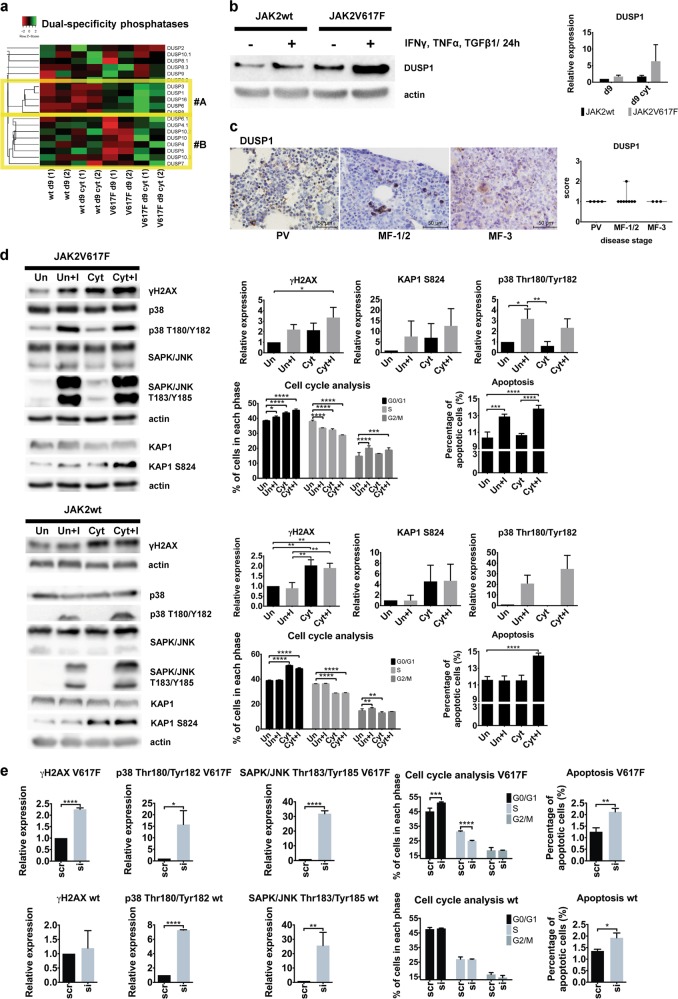
JAK2V617F cell-specific DUSP1 overexpression and DUSP1-dependent proliferation and survival of JAK2V617F cells. **a** Cluster analysis of heatmap representation of differential expression of DUSP genes in d9 and d9 cyt JAK2wt and JAK2V617F^+^ CD34^+^ P-ECs. Columns in the heatmap represent individual samples in experimental duplicates (1, 2). Heatmap was constructed by using Kendall’s Tau Distance Measurement Method and Centroid Linkage Clustering Method. Cluster #A contains intrinsically upregulated DUSPs in d9 and cluster #B contains DUSPs upregulated in d9 cyt JAK2V617F^+^ CD34^+^ P-ECs. **b** Left: Western blotting analysis of DUSP1 in untreated (d9) or treated with IFNγ, TNFα and TGFβ1 for 24 h (d9 cyt) JAK2wt and JAK2V617F^+^ CD34^+^ P-ECs. Right: This chart shows relative expression of DUSP1 in three independent experiments normalized to actin. **c** Representative IHC staining of BM sections for DUSP1 among different disease stages of PV progression. Scale bar, 50 µm. Boxplot shows quantification of the number of cells expressing DUSP1 in sections of patients from grouped disease stages. **d** Western blotting of indicated markers of DDR (γH2AX, KAP1) and SAPK (p38MAPK, JNK) activation, changes in cell cycle profile and percentage of apoptotic cells for HELV617F cells (JAK2V617F; *upper panels*) and HEL-edited cells (JAK2wt; *bottom panels*) untreated (Un) or treated with inflammatory cytokines (Cyt) and without or with exposure to BCI (Un + I or Cyt + I). Charts show γH2AX, Ser 824-phosphorylated KAP1 (KAP1 S824) and p38 T180/Y182 relative expression in three independent experiments normalized to actin. KAP1 S824 is an ATM target site [82]. Cell cycle distribution and apoptosis bar charts show percentage of cell numbers ± SD of four independent experiments in each phase of cell cycle and percentage of apoptotic cells, respectively. *P ≤ 0.05, **P *≤* 0.01, ***P ≤ 0.001, ****P ≤ 0.0001, one-way (western blotting) and two-way (cell cycle and apoptosis) ANOVA. **e** Effect of DUSP1 knock-down in three independent experiments in cytokine-untreated HELV617F and HEL-edited cells on γH2AX and SAPK (p38MAPK, JNK) expression (left graphs), and cell cycle distribution and apoptosis (right graphs). Effects of DUSP1-specific siRNA (si) are compared with scrambled RNA control (scr). Data are normalized and plotted as in **d**. *P ≤ 0.05, **P ≤ 0.01, ***P ≤ 0.001, ****P ≤ 0.0001, two-way ANOVA (cell cycle) and unpaired Student’s *t-*test (two-tailed, the rest). See also Supplementary Fig. 4

We hypothesized that the observed intrinsic resistance of the JAK2V617F^+^ progenitors against inflammation-evoked DNA damage involves stress-activated protein kinases (SAPKs) from mitogen-activated protein kinase (MAPK) superfamily [42]. SAPKs include Jun kinases (JNKs) and p38MAPK that maintain survival of cells exposed to environmental stresses including inflammation [43, 44], however, when p38MAPK activation is too strong, cells including the JAK2V617F^+^ progenitors, undergo apoptosis or senescence [45, 46]. Dual-specificity MAPK phosphatases (DUSPs) support cancer cell survival by buffering SAPKs activities under inflammatory conditions [47]. Some of the DUSPs, including DUSP1, were previously implicated in cellular protection against accumulation of DNA damage and DDR checkpoints [48]. Analysis of *DUSP* gene family revealed mostly upregulated genes in the JAK2V617F^+^ CD34^+^ P-ECs, either intrinsically with further increase in the d9 cyt cultures, or differentially upregulated in the d9 cyt JAK2V617F^+^ cells when compared with the JAK2wt and untreated mutant CD34^+^ P-ECs (Fig. 4a). Among the intrinsically upregulated DUSPs in JAK2V617F^+^ CD34^+^ P-ECs dominated those with substrate specificity for JNK and p38MAPK: DUSP1, DUSP16, and DUSP8 [47]. Recently, JAK2V617F was shown to induce expression of *DUSP1* in a cellular model [49]. In a mouse model of c-Jun-mediated fibrosis, Dusp1 overexpression represented the main negative feedback loop that antagonized p38MAPK/JNK activities [50]. Moreover, *DUSP1*, a known NF-κB target [51], clustered to differentially overexpressed genes in our NF-κB gene set characterizing the network upregulated in the JAK2V617F^+^ CD34^+^ P-ECs (Supplementary Fig. 1k). The DUSP1 protein was highly inducible in the d9 cyt JAK2V617F^+^ CD34^+^ P-ECs (Fig. 4b). Our analyses of clinical patients’ BM biopsies along PV progression revealed moderate DUSP1 positivity in all samples at all PV disease stages (Fig. 4c). DUSP6 was expressed to a much lesser degree in patients’ BM (Supplementary Fig. 4a). Together, these findings indicated a role of DUSP1 in limiting the stress-induced signaling in PV progenitors, thereby preventing a robust activation of cell cycle checkpoints and cellular senescence.

To functionally assess the contribution of high DUSP1 expression in the JAK2V617F^+^ progenitors, we used human erythroleukemia (HEL) cells harboring the *V617F* oncogenic mutation as well as CRISPR/Cas9 JAK2-edited (JAK2wt) HEL cells (see Supplementary Materials and Methods for details on JAK2-edited HEL cells) and exposed them to a DUSP1/6 inhibitor ((E)-2-benzylidene-3-(cyclohexylamino)-2,3-dihydro-1H-inden-1-one; BCI) [52]. Based on preliminary testing (Supplementary Fig. 4b), we chose 1-h BCI treatment to examine the effect of DUSP1/6 inhibition on cell signaling and the fate of unstressed and inflammatory factor-stressed JAK2V617F and JAK2-edited (JAK2wt) HEL cells. We observed more elevated DUSP1 and DUSP6 in the HELV617F cells compared with the HEL-edited JAK2wt cells, however, whereas DUSP6 expression after cytokine treatment sharply decreased, the post-treatment DUSP1 levels increased (Supplementary Fig. 4c). These results suggested that DUSP1 is the main target of the BCI inhibitor in these cells.

As expected, Thr180/Tyr182-phosphorylated p38MAPK (p38 T180/Y182) and Thr183/Tyr185-phosphorylated JNK (JNK T183/Y185) increased after BCI treatment in both HELV617F and HEL-edited cells, regardless of cytokine treatment (Fig. 4d). However, a major difference was observed in cytokine-untreated HEL cells, as γH2AX was largely unaffected in BCI-treated HEL-edited cells, contrary to enhanced γH2AX in the HELV617F cells (Fig. 4d). Non-responsiveness of cytokine-untreated HEL-edited cells to BCI was corroborated by analyses of cell cycle progression and apoptosis. Whereas the increased SAPKs activities after BCI exposure corresponded to significantly more cells in G1 phase and increased apoptosis of the HELV617F cells, the proportions of G1 and apoptotic HEL-edited cells remained unchanged (Fig. 4d). The HEL-edited cells accumulated DNA damage (γH2AX) and increased apoptotic cells only when the BCI exposure was combined with cytokine treatment (Fig. 4d). To confirm that the BCI-inhibitory effects are attributable to DUSP1 inhibition, we knocked down DUSP1 by small interfering RNA (siRNA) in HELV617F and HEL-edited cells (Supplementary Fig. 4d). Indeed, siRNA-mediated depletion of DUSP1 induced changes in protein expression and cell fate phenotypes similar to BCI treatment (Fig. 4e and Supplementary Fig. 4e).

Collectively, all these data indicate that the JAK2V617F^+^ progenitors are strongly dependent on DUSP1 activity for proliferation and survival and that high DUSP1 expression provides adaptation of the JAK2V617F^+^ progenitors to inflammatory stress.

## Discussion

This study contributes to understanding of cancer in general, and pathobiology of PV in particular, at several levels: (i) conceptual; (ii) mechanistic; (iii) clinically related, with therapeutic implications.

From a broader conceptual perspective, our results highlight intriguing differences between the impact of oncogenes activated in common human malignancies, and the JAK2V617F oncogene driving the MPNs including PV. Thus, the former oncogenes, such as Ras, Myc, etc. cause endogenous DNA damage at early stages of tumorigenesis, triggering cell cycle checkpoints and the ensuing cellular senescence or cell death, events that originally led us and others to formulate the concept of DDR as an intrinsic barrier against activated oncogenes and tumor progression of major types of carcinomas [53–55]. This concept, which we have later extended also to brain tumors [56, 57] and *MLL-ENL* oncogene-induced leukemogenesis [58], furthermore explains the disease-evolutionary pressure to select for inactivating mutations in checkpoint genes such as *TP53*, *ATM* or *CHK2* [53–55, 59], as secondary events that allow checkpoint bypass and tumor cell proliferation, at the expense of high genomic instability. In contrast, we report here that such fate is largely prevented in the JAK2V617F-driven PV progenitor cells known to show long-term cell proliferation with a rather stable genome [8, 60]. Our data suggest that this unorthodox fate of PV cells reflects the lack of any robust endogenous DNA damage or checkpoint activation, despite the complex PV-associated inflammatory and oxidative stresses that are known to trigger pronounced DDRs and cellular senescence or death in other inflammation-associated settings [18, 38, 61]. As explained below, we propose that the underlying mechanisms for such unique pathobiological behavior reflect features “in-built” among the effects of the JAK2V617F oncogene, effects that mitigate the overall extent and/or impact of the inflammatory and oxidative insults in PV progenitors, thereby allowing for chronic proliferation in the indolent phase of JAK2V617F^+^ MPNs [8, 60].

Some studies including in vitro model experiments and cultured patients’ cells, suggested increased DNA damage in the JAK2V617F hematopoietic compartment [32, 33, 62], a result at odds with our present dataset. However, if the PV JAK2V617F cells indeed accumulated high ROS-dependent oxidative stress [32] or featured enhanced DNA breakage [33], the proliferative stage of the disease would be shorter and compromised by checkpoint activation, events that do not match the known disease course or our results. Here, we identified some mechanistic aspects downstream of JAK2V617F that explain the apparent discrepancies and the unique behavior of the PV progenitors. First, using complementary models and approaches, we identified DUSP1 phosphatase as a candidate factor that mediates signaling adaptation to inflammatory stress in JAK2V617F^+^ PV progenitors. DUSP1 counteracts JNK and p38MAPK activities [47], the key kinases activated in stressed hematopoiesis, including inflammation-exposed HSCs [7, 44]. We found DUSP1 upregulated downstream of the JAK2V617F-stimulated NF-κB pathway, consistently with earlier study [51]. Notably, we report that DUSP1 is highly expressed specifically in JAK2V617F^+^ CD34^+^ P-ECs, in HELV617F cells and in the PV patients’ BM. Furthermore, our experimental chemical inhibition as well as siRNA-mediated knock-down of DUSP1 leads to JNK/p38MAPK reactivation, accumulation of DNA damage (γH2AX) and accelerated cell cycle arrest and apoptosis of JAK2V617F^+^, but not JAK2wt cells. These data indicate that JAK2V617F^+^ progenitors depend on DUSP1 activity for proliferation and survival, and that elevated DUSP1 provides adaptation of JAK2V617F^+^ progenitors to inflammatory stress. Another important aspect of our data in this context is the evidence for a hierarchical cytokine-induced inflammatory network in JAK2V617F^+^ CD34^+^ P-ECs, including intrinsic IFNγ priming fueled by JAK2V617F. As a consequence of the specific JAK2V617F-mediated inflammatory phenotype with ensuing potentially genotoxic ROS, we identified a hyper-activated “adaptive” ROS-buffering system. Given that DUSP activity is sensitive to ROS-mediated oxidation [63], our results suggest that upregulation of the antioxidant defense driven by JAK2V617F is required for enhanced DUSP1 activity. We propose that cooperation between high antioxidant buffering capacity and DUSP1 activity form a regulatory loop that allows proliferation and survival of JAK2V617F^+^ progenitors by protecting them against endogenous DNA damage. Another complementary factor that can help avoid extensive DNA damage is RECQL5, a JAK2V617F/STAT-upregulated helicase that mitigates replication stress and thereby helps maintain genomic stability in the chronic phase of MPN [34].

Last but not least, in the clinical context, our dataset has implications for understanding the disease course and potentially innovative treatment. Thus, our model experiments are consistent with our biomarker analyses of patients’ BM biopsies from diverse stages of PV. Our mechanistic data also suggest that with increasing risk of fibrotic transformation, associated with enhanced inflammation and ROS production [4, 64], the “protective” DUSP1 activity gets gradually suppressed, resulting in sustained JNK/p38MAPK activation and ensuing expression of inflammatory mediators, promoting fibrotic transformation [65], dysmegakaryopoiesis [10], and anemia associated with MF [66]. In summary, our results suggest that DUSP1 addiction in the early stages of PV offers not only insights into the unique pathobiology, but also a potential therapeutic target, as inhibition of DUSP1 leading to JNK/p38MAPK hyperactivation may provide a strategy for elimination of the cycling JAK2V617F^+^ progenitors. Given that JAK2V617F^+^ progenitors maintain a relatively high ROS status, synthetic lethality-based approach can be exploited to selectively target these cells by escalating the oxidative stress using MTH1 inhibitors [67] and simultaneously target DUSP1 for DNA damage elevation to the level inducing cell death.

## Materials and methods

### Culture of undifferentiated iPSCs

Patient-specific iPSC line with JAK2V617F genotype (PVB1.4, obtained from original investigators J.T. Prchal and L. Cheng) was described in detail previously [12, 68]. Control iPSC line with wild-type JAK2 genotype (BXS0116) was obtained from ATCC and its characterization including capacity to activate DDR was published elsewhere [13]. iPSCs were cultured as described [12]. For radiation experiments, cells were irradiated with 2 Gy of X-rays (RS Research Cabinet, Xstrahl, Surrey, UK) and incubated 3 h before harvesting.

### iPSC hematopoietic differentiation

CD34^+^ P-ECs with hematopoietic differentiation program were generated from iPSCs using published protocol [14]. For cytokines used and workflow of iPSCs differentiation see Supplementary Materials and Methods. To test cytokine effects, IFNγ (100 U/ml), TNFα (50 ng/ml), and TGFβ1 (5 ng/ml) were added to culture media 24 h prior to harvesting cells for analysis (day 9 of differentiation; all cytokines purchased from ProSpec-Tany TechnoGene, Rehovot, Israel).

### Generation of JAK2-edited (JAK2wt) HEL cell line

JAK2V617F mutation in HEL cells (obtained from DSMZ—German Collection of Microorganisms and Cell Cultures) was repaired by HR using CRISPR/Cas9 (CRISPR construct pXPR001; Addgene #49535) with inserted sgRNA (5ʹ- ACGAGAGTAAGTAAAACTAC-3ʹ) and long dsDNA template spanning *JAK2* exon 24 (1818 bp). HEL cells were electroporated using Amaxa™ Nucleofector™ (kit V, program X-005), single-cell sorted 48 h post electroporation based on their fluorescence status and single-cell clones expanded. Functional comparison of HEL-edited (JAK2wt) with control parental (unedited HELV617F) clones is described in Supplementary Materials and Methods.

### Microarray and data analysis

Two biological replicates per group were used. Total RNA was isolated using TRI Reagent (Sigma-Aldrich) and RNA integrity tested by Agilent 2100 Bioanalyzer (RNA integrity number > 9; Agilent Technologies). RNA was amplified and labeled using Illumina TotalPrep RNA Amplification Kit (Ambion; Thermo Fisher) and hybridized on HumanHT-12 v4 Expression BeadChips (Illumina). Raw data were processed using Genome Studio software (version 1.9.0.24624; Illumina) and analyzed within the oligo and limma packages of the Bioconductor [69–71]. Moderated *t*-test was used to detect DEGs between the sample groups: at least twofold change in gene expression and Storey’s *q*-value <0.1 were required [72]. MIAME compliant data were deposited to the Array Express database (E-MTAB-7693). Heatmapper tool was used to generate heatmaps to visualize differences in gene expression [73]. Heatmaps were constructed with Pearson Distance Measurement Method and Average or Centroid Linkage Clustering Method.

### Pathway over-representation analysis and GSEA

Over-representation analysis of DEGs and generation of interaction network module maps using gene regulatory interactions was carried out with the ConsensusPathDB software (Release 32 (11.01.2017)) using 12 biological pathways databases (Wikipathways, Inoh, Netpath, Smpdb, Pharmgkb, Biocarta, Kegg, Reactome, Humancyc, Signalink, Ehmn, and Pid) to identify pathways associated with the mRNAs differentially expressed in studied samples [74]. GSEAs were performed with GSEA v3.0 software [75]. Ratio_of_Classes was used as a metric to rank genes.

### Quantitative real-time polymerase chain reaction

Total RNA was reverse transcribed (Roche) and treated with TURBO DNA-free DNase (Thermo Fisher). LightCycler 480 platform (Roche) was employed as described [76], using *RPLP0* as a reference gene. Primers are listed in Supplementary Table 1.

### Flow cytometry analysis

Flow cytometry analysis was done using Cytomics FC500 and CXP software (Beckman Coulter). For cell cycle distribution analysis and apoptosis, CD34^+^ P-ECs were treated as described in Supplementary Fig. 3a and then labeled with 10 µM BrdU (Sigma-Aldrich). Combined BrdU and propidium iodide staining was as described [77], gating strategy is shown in Supplementary Materials and Methods. ROS production was measured by CellROX Green Flow Cytometry Assay Kit in CD34^+^ P-ECs treated with defined combinations of cytokines for 3, 6, 12, and 24 h. The surface markers CD34, CD41, and CD43 were detected with the respective antibodies using dilutions specified in Supplementary Table 2. In all experiments, appropriate isotype control antibodies were used.

### Enzyme assay

The activity of antioxidative enzymes was determined according to standard methods [78, 79]. The enzyme activity was expressed in units per gram of total protein. The total antioxidant capacity was determined using the Total Antioxidant Capacity Assay Kit (Sigma-Aldrich).

### Immunocytochemistry on EBs in agarose gel matrix saturated with paraffin

Paraffin-embedded JAK2wt and JAK2V617F^+^ EBs were prepared as described [80]. Nonspecific binding sites were blocked with 0.2% bovine serum albumin for 1 h, sections stained with primary antibody at RT for 1 h. EnVisionTM system (Dako, Glostrup, Denmark) including horseradish peroxidase and diaminobenzidine (DAB^+^, Dako) was used for antibody detection; cell nuclei were counterstained by hematoxylin. Images were acquired using an Olympus BX51 inverted microscope equipped with ColorViewIII digital CCD camera.

### Immunocytochemistry of CD34^+^ P-ECs

Single-cell suspension from CD34^+^ P-EC was cytospined onto microscope slides, cells fixed by 3% formaldehyde, permeabilized and stained with respective antibodies listed in Supplementary Table 2.

### Inhibition of DUSPs

To test effects of DUSP1/6 inhibitor (BCI-CAS 15982-84-0, Merck), untreated and cytokine-treated HEL cells were incubated with 10 µM BCI (dissolved in DMSO) for defined times (0.5, 1, 2, 4, and 6 h), in case of cytokine-treated cells prior the end of 24-h cytokine treatment.

### DUSP1 silencing by siRNA

Control parental HEL cells and HEL-edited cells were transfected with validated Silencer Select siRNA (Thermo Fisher Scientific, Assay ID: s4363) and with corresponding recommended Silencer Select siRNA-negative control (scr) using RNAiMAX transfection reagent (Thermo Fisher Scientific). Transfected cells were subsequently harvested at 24, 48, and 72 h after transfection for RNA and 72 h for protein lysates.

### IHC of patients’ samples

Formalin-fixed, paraffin-embedded tissue samples were sectioned and processed using standard protocols for biopsy processing. BM trephine biopsies were demineralized by 10% chelatone 3 (pH 8) directly after fixation. To grade fibrosis, Gomori’s silver impregnation for reticulin was performed. Visualization of antigens is described in Supplementary Materials and Methods, with antibodies specified in Supplementary Table 2. For staining evaluation, published scoring was chosen [81], and the results presented as boxplots where each dot represents a patient’s sample plotted against the staining score. The whiskers indicate samples within the minimum and maximum values and the line across the box represents median. More details are in Supplementary Materials and Methods.

### Patients’ samples

Human BM samples were obtained from the Department of Clinical and Molecular Pathology, University Hospital Olomouc (UHOL), Olomouc, Czech Republic. The original examinations were obtained with the approval of the IRB committee of the UHOL and according to the Declaration of Helsinki. Additional information on patient samples is available in Supplementary Table 3.

### Statistical analysis

Mean values ± SD are shown. Mann–Whitney test, Student’s unpaired two-tailed *t*-test, two-way and one-way ANOVA with Bonferroni correction for multiple comparisons were used for obtaining statistical significance values (using Graph-Pad Prism Version 6.0).

## Supplementary information

Supplementary Figure Legends

Supplementary Figure 1

Supplementary Figure 2

Supplementary Figure 3

Supplementary Figure 4

Supplementary Materials and Methods
