# Supplementary material for: Addiction to DUSP1 protects JAK2V617F-driven polycythemia vera progenitors against inflammatory stress and DNA damage, allowing chronic proliferation
Source: Oncogene. 2019 Apr 9;38(28):5627–42. doi: 10.1038/s41388-019-0813-7 (PMC6756199; doi:10.1038/s41388-019-0813-7)
Supplement: Supplementary file 1 — Supplementary Figure Legends [file 41388_2019_813_MOESM1_ESM.doc]

**Supplementary Figure 1 (Related to Figure 1):** Characterization of differentiation status of CD34^+^ P-ECs, DDR capacity of JAK2wt and JAK2V617F^+^ iPSCs**,** transcriptome profiles of JAK2wt and JAK2V617F^+^ CD34^+^ P-ECs and PV patients' BM staining for pro-inflammatory cytokines. **a** Comparable cell populations defined by CD34, CD41 and CD43 antigens positivity analyzed by flow cytometry in d9 JAK2wt and JAK2V617F^+^ CD34^+^ P-ECs, presented as means and SD values for the percentage of positive cells for individual surface antigens. **b** Gating strategy for the assessment of CD34, CD41 and CD43 surface antigens by flow cytometry with isotype controls non-specific background exclusion. **c** Analysis of normalized microarray expression data for early hematopoietic markers showing hallmarks of very early hematopoiesis defined by KIT and CD34 upregulation and THY1 downregulation upon differentiation of JAK2wt and V617F^+^ iPSC lines into d9 CD34^+^ P-ECs. **d** Heatmap showing expression of gene set members for signaling pathways regulating pluripotency of stem cells (KEGG, n = 139) with most of the genes in clusters with similar expression patterns between d9 JAK2wt and JAK2V617F^+^ CD34^+^ P-ECs (d9) upon differentiation from iPSCs (d0). Enlarged clusters (*right*) highlight genes downregulated over the course of differentiation. Columns in the heatmap represent individual samples in experimental duplicates (1, 2). **e** Venn diagrams and source tables showing overlapping and unique differentially upregulated genes (*left*) and differentially downregulated genes (*right*) in KEGG pathway: Hematopoietic cell lineage (n = 97) upon differentiation of JAK2wt and V617F^+^ iPSC lines (d0) into d9 CD34^+^ P-ECs (d9) with logFC > 0.5 and adjusted p-value < 0.05 for target genes cut-off. **f** Immunoblotting analysis of total and phosphorylated forms of p53, Chk1 and Chk2 in JAK2wt and V617F^+^ iPSCs, which were assayed before (Un) and after X-ray irradiation (X). **g** Immunocytochemical dual staining against IFNγ and CD34 in paraffin-embedded JAK2wt (wt) and V617F^+^ (V617F) EBs counterstained by DAPI. Negative control (NC) describes sample without primary antibodies incubation used for setup of exposure time to eliminate background fluorescence. **h** Western blotting analysis of both total and phosphorylated form of STAT1 at Tyr701 in JAK2wt and V617F^+^ CD34^+^ P-ECs, untreated or treated with various combinations of inflammatory cytokines. **i** Tables list top twenty differentially expressed genes (DEGs), ranked according to adjusted p-value, between d9 and d9 cyt JAK2wt (*left* table in black) or JAK2V617F^+^ (*right* table in gray) CD34^+^ P-ECs. List of genes was created using logFC > 1, q < 0.1 cut-off. Expression and adjusted p-values of shared DEGs from both tables are depicted by accompanied bar chart showing higher expression of all shared DEGs in JAK2V617F^+^ CD34^+^ P-ECs. **j** Overrepresentation analysis of differentially upregulated (*left*) and downregulated (*right*) genes (logFC < -1, q < 0.1 cut-off) between d9 and d9 cyt JAK2wt and V617F^+^ CD34^+^ P-ECs compared to listed biological pathways using ConsensusPathDB with minimum overlap of 4 genes in pathway and p-value cut-off of 0.001. **k** Heatmap analysis of NF-κB target genes expression distributed to categories based on Gilmore Lab reference list (n = 424; http://www.bu.edu/nf-kb/gene-resources/target-genes/). **l** IHC staining of TNFα, TGFβ1, CCL3 and IL6 in representative BM sections from patients along the progression of PV. Scale bar, 50 µm and 20 µm. Boxplots show quantification of the number of cells expressing individual cytokines in sections of patients from grouped disease stages. Insets in CCL3 staining show magnification of main CCL3 producers, individual basophile-like cells with segmented nuclei.

**Supplementary Figure 2 (Related to Figure 2):** PV progenitors are refractory to inflammation-evoked DNA damage and exhibit suppressed DDR and DNA repair signatures. **a** Representative IHC staining of BM sections for 8-oxoG along the progression of PV. Scale bar, 20 µm. Boxplots show quantification of the number of cells expressing 8-oxoG from grouped disease stages. **b** Immunocytochemistry staining for γH2AX (red) and RAD51 (green) of d9 JAK2wt and JAK2V617F^+^ CD34^+^ P-ECs, either untreated (Un) or treated with various combinations of inflammatory cytokines. Charts show percentage of cells ± SD with more than five γH2AX or RAD51 foci per cell in nucleus in three independent experiments. **P≤0.05, **P ≤ 0.01*, ****P ≤ 0.001,* two-way ANOVA*.* **c** Conditioned medium (CM) from d9 JAK2V617F^+^ CD34^+^ P-ECs (V617F d9) was harvested and used for treatment of JAK2wt CD34^+^ P-ECs (wt d9) for 24 h. Bar charts and before-after graphs present percentages of cells ± SD with more than five γH2AX or RAD51 foci per cell in nucleus in three independent experiments. **d** Microarray expression data based heatmaps show expression of DDR genes (n = 369 genes) and associated DDR categories.^36^ Heatmaps present samples in experimental duplicates (1, 2), either JAK2wt (wt) or JAK2V617F^+^ (V617F) in all experimental conditions (d9 – untreated d9 CD34^+^ P-ECs; d9 cyt – d9 CD34^+^ P-ECs treated by inflammatory cytokines). Clusters highlighted by blue boxes denote transcripts of genes upregulated in JAK2V617F^+^ CD34^+^ P-ECs, while yellow boxes show transcripts downregulated in JAK2V617F^+^ CD34^+^ P-ECs. **e** Immunocytochemistry staining of untreated (d9 Un) and X-ray irradiated JAK2wt and V617F^+^ CD34^+^ P-ECs (d9 X) stained for γH2AX (red), CHK2 T68 (red) and pATMS1981 (green) counterstained with DAPI (blue). Charts show relative expression of γH2AX, Chk2 T68 and pATMS1981 in representative experiments based on the level of corrected total fluorescence in a nucleus (CTFN). CTFN was calculated as integrated density proportional to fluorescence signal in nucleus subtracted by area of selected nucleus x mean fluorescence of background readings. **f** Western blotting showing levels of γH2AX in untreated (Un) and X-ray irradiated (X) JAK2wt and JAK2V617F^+^ CD34^+^ P-ECs. Chart show relative expression of γH2AX with both JAK2wt and JAK2V617F Un set to one as these were separate individual analyses.

**Supplementary Figure 3 (Related to Figure 3):** Schematics of cell cycle synchronization of CD34^+^ P-EC cells and increased expression and activity of enzymes involved in ROS buffering system in JAK2V617F^+^ CD34^+^ P-ECs. **a** Schematics of time course for cell cycle synchronization with growth factors (GFs) medium depletion, followed by dual pulse labelling with propidium iodide and BrdU incorporation. **b** *Left*: Heatmap representation of antioxidant defense system gene set (n = 33; https://www.qiagen.com/ca/shop/pcr/primer-sets/rt2-profiler-pcr-arrays/?catno=PAHS-065Z#geneglobe)^74^ expression in d9 and d9 cyt JAK2wt and JAK2V617F^+^ CD34^+^ P-ECs. Columns represent individual samples in experimental duplicates (1, 2). *Right*: Bar graphs show glutathione reductase (GR), glucose-6-phosphate dehydrogenase (G6PD), hexokinase (HK) and glutathione peroxidase (GPx) enzyme activities of untreated (d9) and inflammatory cytokine treated (d9 cyt) JAK2wt and V617F^+^ CD34^+^ P-ECs in day 9 of differentiation. Values are presented as a means of enzyme activity ± SD. **P ≤ 0.05, **P ≤ 0.01, ***P ≤ 0.001, ****P ≤ 0.0001,* two-way ANOVA*.*

**Supplementary Figure 4 (Related to Figure 4):** DUSP6 expression in patients' samples and effect of DUSP1/6 inhibition on mitogen-activated protein kinase pathways mediated by p38MAPK and JNK. **a** IHC staining of DUSP6 in representative BM sections along the progression of PV. Scale bar, 50 µm. Boxplots show quantification of positive cell numbers. **b** Western blotting analysis of γH2AX and Thr 180/Tyr 182-phosphorylated p38MAPK (p38 T180/Y182) in HELV617F (JAK2V617F) and HEL-edited cells (JAK2wt), either untreated or treated with inflammatory cytokines (cyt) upon exposure to 10 µM DUSP 1/6 inhibitor in various time points. Charts show relative expression of indicated markers normalized to actin in defined time points. **c** Western blotting analysis of DUSP1 and DUSP6 expression in HELV617F (JAK2V617F) and HEL-edited cells (JAK2wt), either untreated (un) or treated with inflammatory cytokines (cyt). Charts show DUSP1 and DUSP6 relative expression normalized to actin (mean ± SD, n = 3 independent experiments). **P ≤ 0.05, **P ≤ 0.01,* two-way ANOVA. **d** *Left*: Downregulation of *DUSP1* mRNA levels 24, 48 and 72 h after DUSP1 siRNA transfection of HELV617F (V617F) and HEL-edited (wt) cells. *Right*: Decrease of DUSP1 protein level 72 h after DUSP1 siRNA transfection. Effects of DUSP1-specific siRNA (si) are compared to scrambled RNA control (scr). **e** Western blotting of indicated markers of DDR (γH2AX, KAP1) and SAPK (p38MAPK, JNK) activation for HELV617F cells (JAK2V617F; left panels) and HEL-edited cells (JAK2wt; right panels) targeted with DUSP1-specific siRNA (si) and scrambled RNA control (scr), 72 h after siRNA transfection.
