# Supplementary material for: Addiction to DUSP1 protects JAK2V617F-driven polycythemia vera progenitors against inflammatory stress and DNA damage, allowing chronic proliferation
Source: Oncogene. 2019 Apr 9;38(28):5627–42. doi: 10.1038/s41388-019-0813-7 (PMC6756199; doi:10.1038/s41388-019-0813-7)
Supplement: Supplementary file 2 — Supplementary Figure 1 [file 41388_2019_813_MOESM2_ESM.pdf]

a

b

c

d

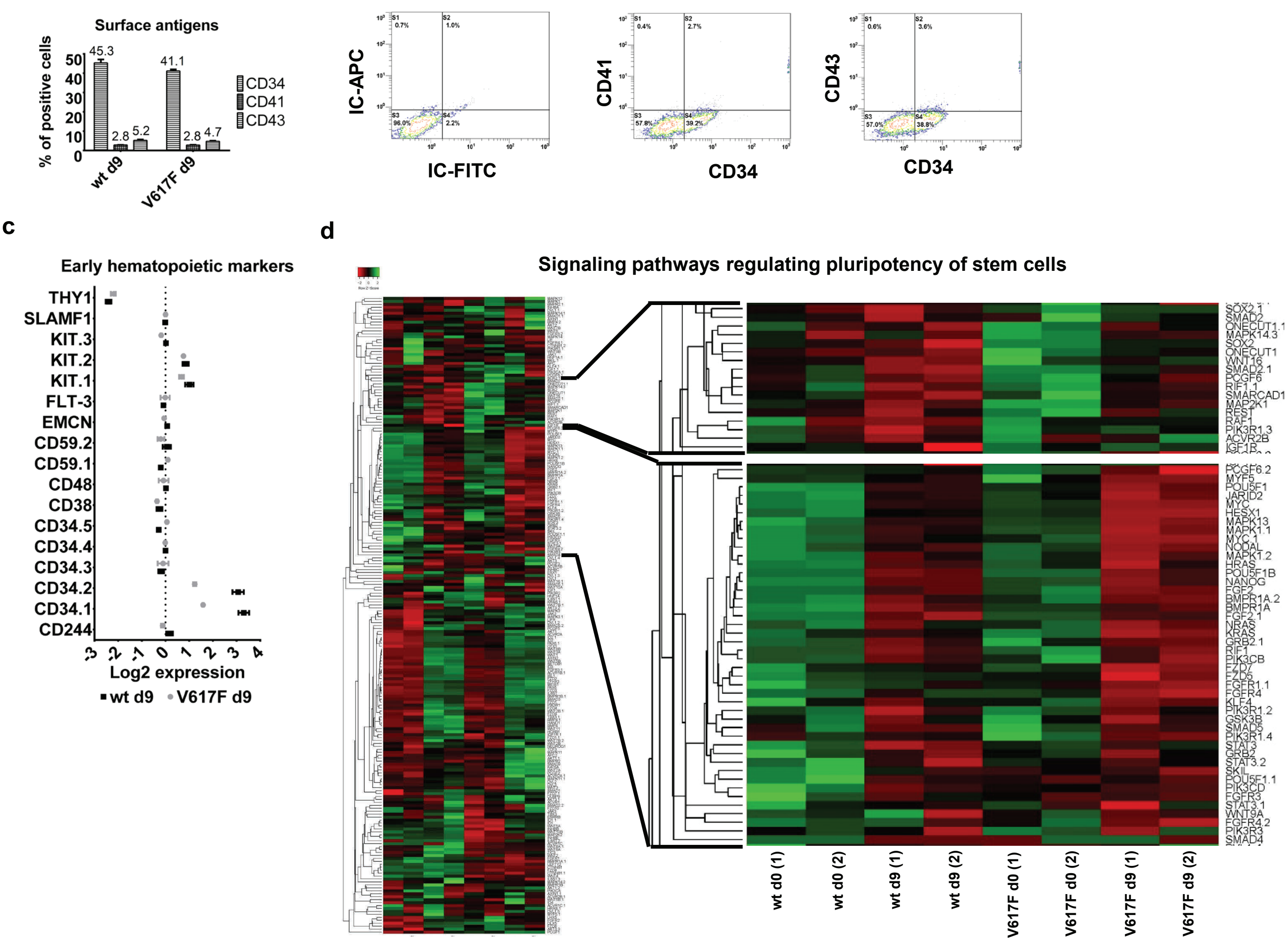

e

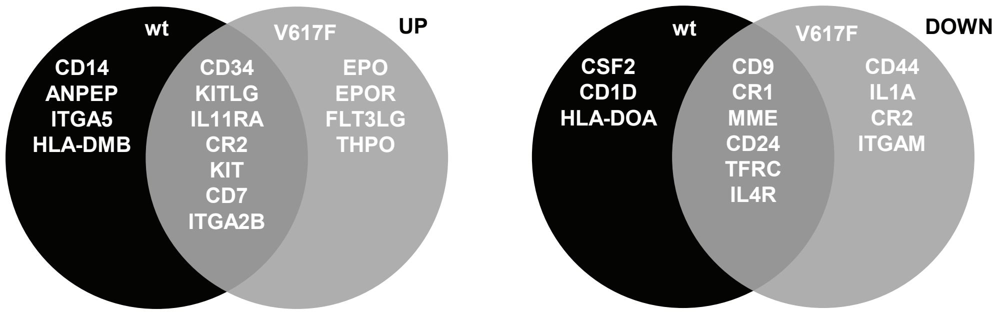

Rank	Symbol	logFC	Adjusted p-Value
1	CD34	3.30	6.14E-07
2	CD34	3.06	3.05E-05
3	KITLG	2.83	6.28E-07
4	KITLG	1.69	5.75E-04
5	IL11RA	1.54	5.40E-05
6	IL11RA	1.53	1.19E-05
7	CD14	1.47	3.82E-04
8	CR2	1.47	8.25E-04
9	ANPEP	1.31	1.22E-03
10	ITGA5	1.15	2.40E-05
11	HLA-DMB	1.02	4.43E-03
12	KIT	1.01	3.88E-04
13	CD7	0.95	2.66E-03
14	KIT	0.85	2.89E-03
15	ITGA2B	0.52	4.87E-02

Rank	Symbol	logFC	Adjusted p-Value
1	KITLG	2.07	5.61E-06
2	KITLG	1.69	6.38E-04
3	CR2	1.68	4.05E-04
4	CD34	1.59	5.63E-05
5	IL11RA	1.24	5.63E-05
6	CD34	1.23	8.33E-03
7	IL11RA	1.01	8.84E-04
8	KIT	0.764	5.45E-03
9	EPO	0.734	2.12E-02
10	ITGA2B	0.727	9.92E-03
11	KIT	0.671	4.57E-03
12	EPOR	0.633	1.13E-02
13	FLT3LG	0.621	4.06E-02
14	CD7	0.541	4.68E-02
15	THPO	0.538	4.55E-02

Rank	Symbol	logFC	Adjusted p-Value
1	CD9	-2	1.11E-05
2	CR1	-1.35	1.55E-04
3	MME	-1.25	6.87E-05
4	CSF2	-1.07	4.82E-03
5	CD1D	-1	1.38E-02
6	CD24	-0.854	3.70E-03
7	TFRC	-0.723	8.08E-03
8	IL4R	-0.693	5.29E-03
9	HLA-DOA	-0.644	1.92E-03
10	CD24	-0.627	2.10E-03

Rank	Symbol	logFC	Adjusted p-Value
1	MME	-1.98	6.15E-06
2	CD9	-1.92	2.02E-05
3	CD44	-1.82	2.65E-05
4	IL1A	-1.63	2.65E-05
5	CD24	-1.31	3.16E-04
6	CR1	-1.02	1.04E-03
7	CR2	-0.786	4.54E-02
8	ITGAM	-0.782	1.29E-02
9	CD24	-0.754	7.54E-04
10	IL4R	-0.648	7.78E-03
11	TFRC	-0.514	4.32E-02

f

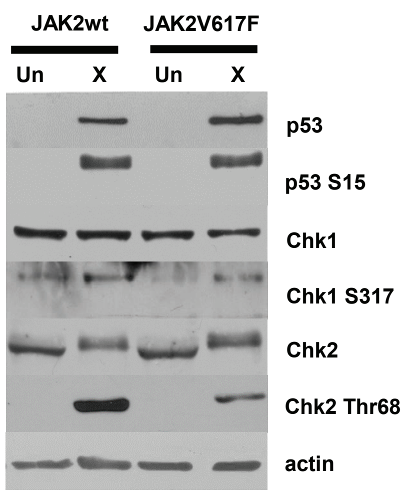

g

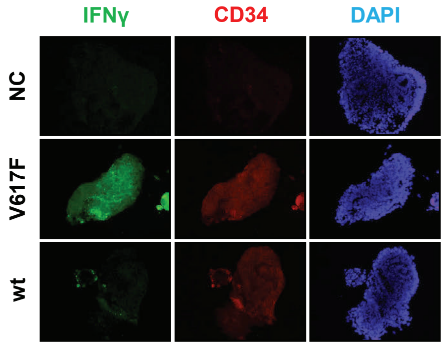

h

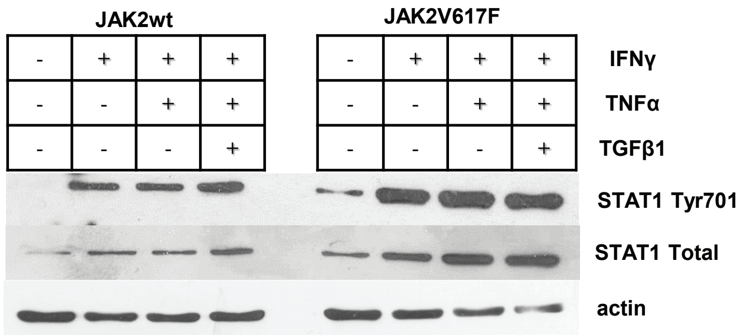

i

DEGs: JAK2wt d9 vs. d9 cyt			
Rank	Symbol	logFC	Adjusted p-Value
1	TAP1	2.46	1.90E-05
2	CCL2	2.18	3.02E-04
3	IRF1	1.59	4.72E-03
4	OPTN	1.52	4.72E-03
5	UBD	3.37	4.72E-03
6	IL32	1.7	4.72E-03
7	CXCL10	2.43	4.72E-03
8	B2M	1.32	4.72E-03
9	IL32	2.26	4.72E-03
10	VCAM1	1.49	4.72E-03
11	LTB	2.53	9.90E-03
12	NODAL	1.39	9.90E-03
13	PLA2G4C	1.14	9.90E-03
14	FGA	-1.54	9.90E-03
15	SPACA6	1.09	1.08E-02
16	STAT1	1.23	1.21E-02
17	STAT1	1.01	2.02E-02
18	PARP9	1.16	2.24E-02
19	RARRES3	1.52	2.86E-02
20	CD70	1.13	4.02E-02

DEGs: JAK2V617F d9 vs. d9 cyt			
Rank	Symbol	logFC	Adjusted p-Value
1	GBP1	4.01	7.52E-07
2	CXCL10	6.73	1.18E-06
3	TAP1	2.94	1.18E-06
4	VCAM1	2.38	3.70E-06
5	VCAM1	3.26	5.20E-06
6	CSAG3	2.4	5.20E-06
7	IL32	4.41	9.61E-06
8	CCL2	2.67	9.61E-06
9	IRF1	2.79	9.61E-06
10	GBP1	3.49	9.61E-06
11	RARRES3	3.74	1.36E-05
12	GBP2	2.85	2.67E-05
13	UBD	5.35	2.76E-05
14	IL32	2.63	3.57E-05
15	STAT1	2.04	3.84E-05
16	PSMB9	3.28	4.05E-05
17	B2M	2.05	4.08E-05
18	STAT1	2.2	5.64E-05
19	SERPINA3	3.25	9.43E-05
20	HCP5	1.37	1.04E-04

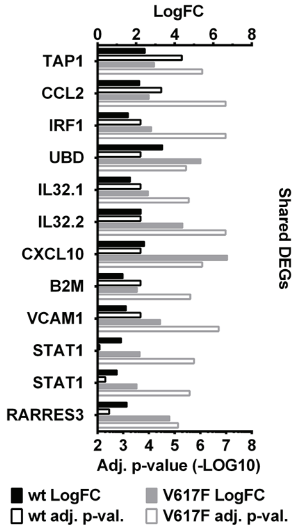

j

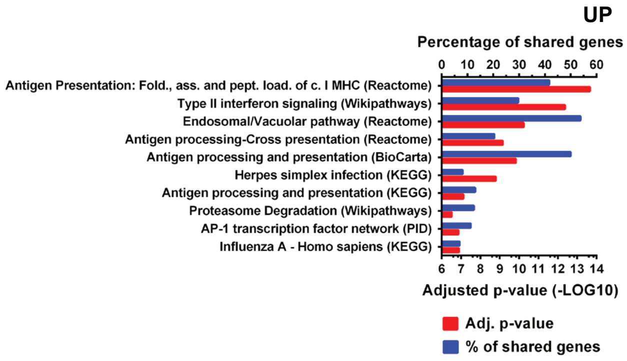

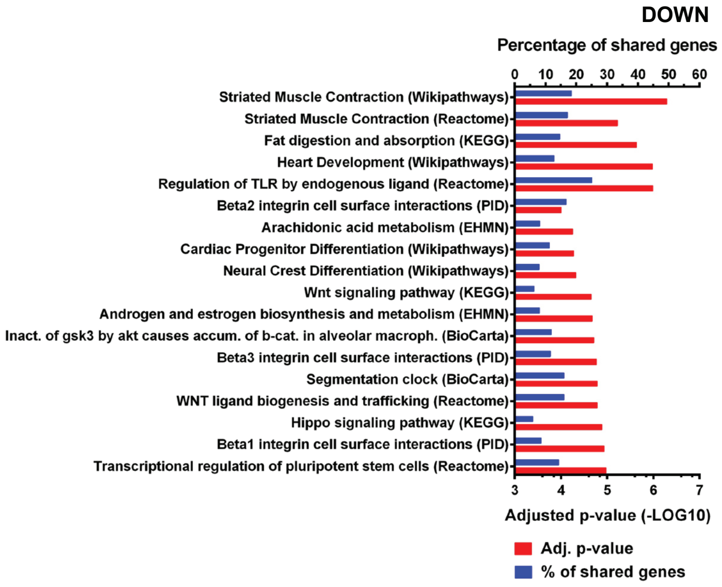

k

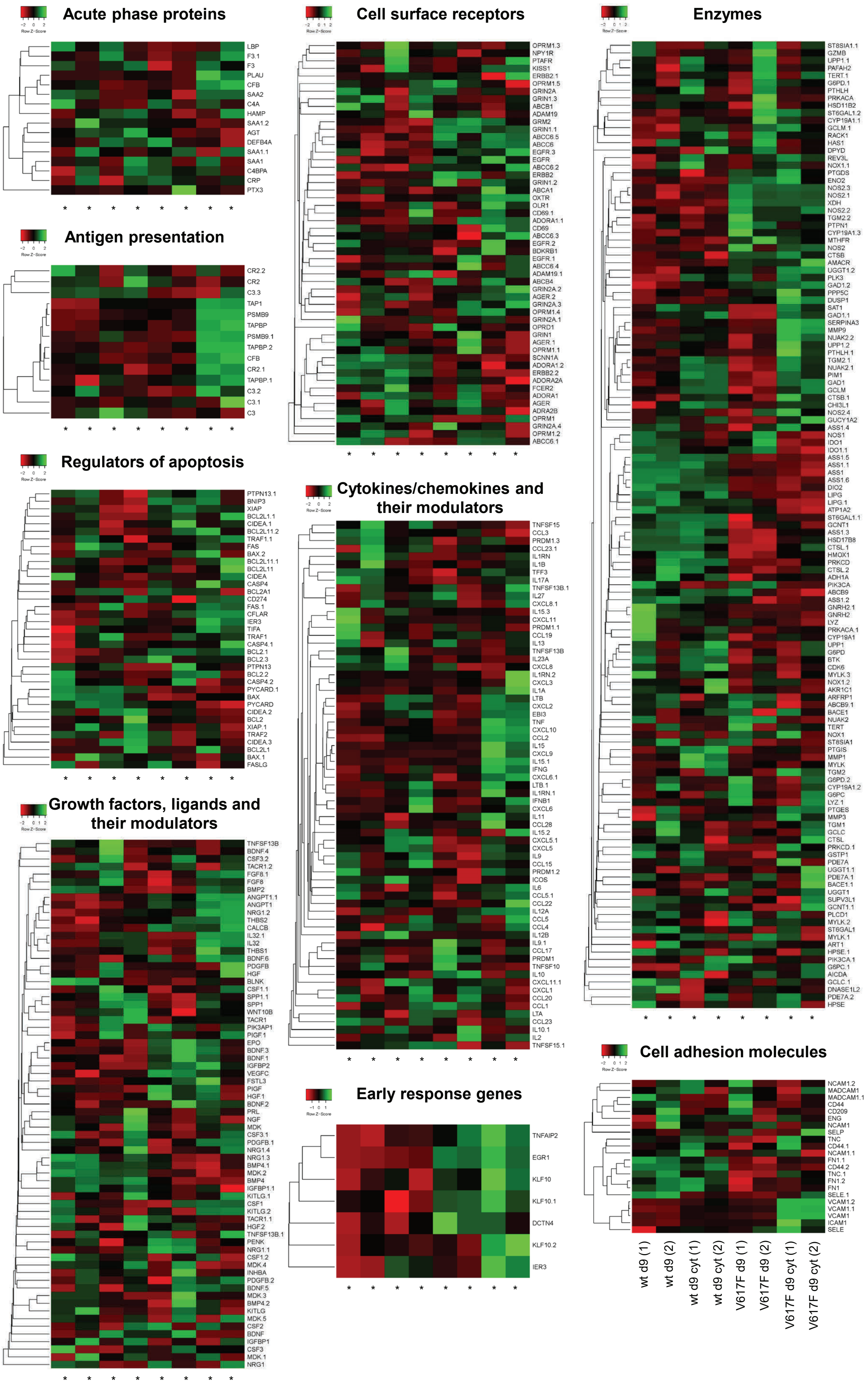

Acute phase proteins
LBP
F3.1
F3
PLAU
CFB
SAA2
C4A
HAMP
SAA1.2
AGT
DEFB4A
SAA1.1
SAA1
C4BPA
CRP
PTX3
Antigen presentation
CR2.2
CR2
C3.3
TAP1
PSMB9
TAPBP
PSMB9.1
TAPBP.2
CFB
CR2.1
TAPBP.1
C3.2
C3.1
C3
Regulators of apoptosis
PTPN13.1
BNIP3
XIAP
BCL2L1.1
CIDEA.1
BCL2L11.2
TRAF1.1
FAS
BAX.2
BCL2L11.1
BCL2L11
CIDEA
CASP4
BCL2A1
CD274
FAS.1
CFLAR
IER3
TIFA
TRAF1
CASP4.1
BCL2.1
BCL2.3
PTPN13
BCL2.2
CASP4.2
PYCARD.1
BAX
PYCARD
CIDEA.2
BCL2
XIAP.1
TRAF2
CIDEA.3
BCL2L1
BAX.1
FASLG
Growth factors, ligands and their modulators
Cell surface receptors
OPRM1.3
NPY1R
PTAFR
KISS1
ERBB2.1
OPRM1.5
GRIN2A
GRIN1.3
ABCB1
ADAM19
GRM2
GRIN1.1
ABCC6.5
ABCC6
EGFR.3
EGFR
ABCC6.2
ERBB2
GRIN1.2
ABCA1
OXTR
OLR1
CD69.1
ADORA1.1
CD69
ABCC6.3
EGFR.2
BDKRB1
EGFR.1
ABCC6.4
ADAM19.1
ABCB4
GRIN2A.2
AGER.2
GRIN2A.3
OPRM1.4
GRIN2A.1
OPRD1
GRIN1
AGER.1
OPRM1.1
SCNN1A
ADORA1.2
ERBB2.2
ADORA2A
FCER2
ADORA1
AGER
ADRA2B
OPRM1
GRIN2A.4
OPRM1.2
ABCC6.1
Cytokines/chemokines and their modulators
Early response genes
TNFAIP2
EGR1
KLF10
KLF10.1
DCTN4
KLF10.2
IER3
Enzymes
Cell adhesion molecules
NCAM1.2
MADCAM1
MADCAM1.1
CD44
CD209
ENG
NCAM1
SELP
TNC
CD44.1
NCAM1.1
FN1.1
CD44.2
TNC.1
FN1.2
FN1
SELE.1
VCAM1.2
VCAM1.1
VCAM1
ICAM1
SELE
wt d9 (1)
wt d9 (2)
wt d9 cyt (1)
wt d9 cyt (2)
V617F d9 (1)
V617F d9 (2)
V617F d9 cyt (1)
V617F d9 cyt (2)

k

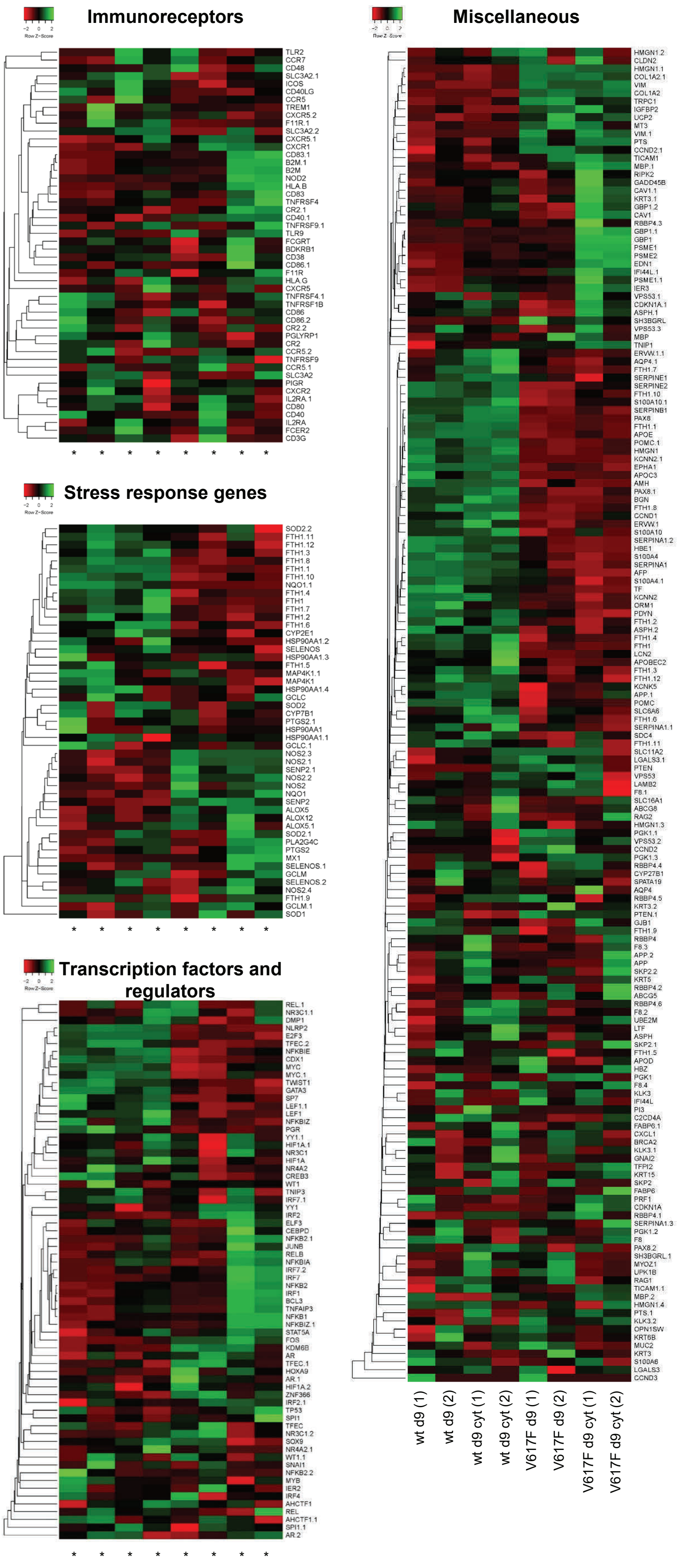
Immunoreceptors
Stress response genes
Transcription factors and regulators
Miscellaneous
Row Z-Score
-2 0 2
wt d9 (1)
wt d9 (2)
wt d9 cyt (1)
wt d9 cyt (2)
V617F d9 (1)
V617F d9 (2)
V617F d9 cyt (1)
V617F d9 cyt (2)

I

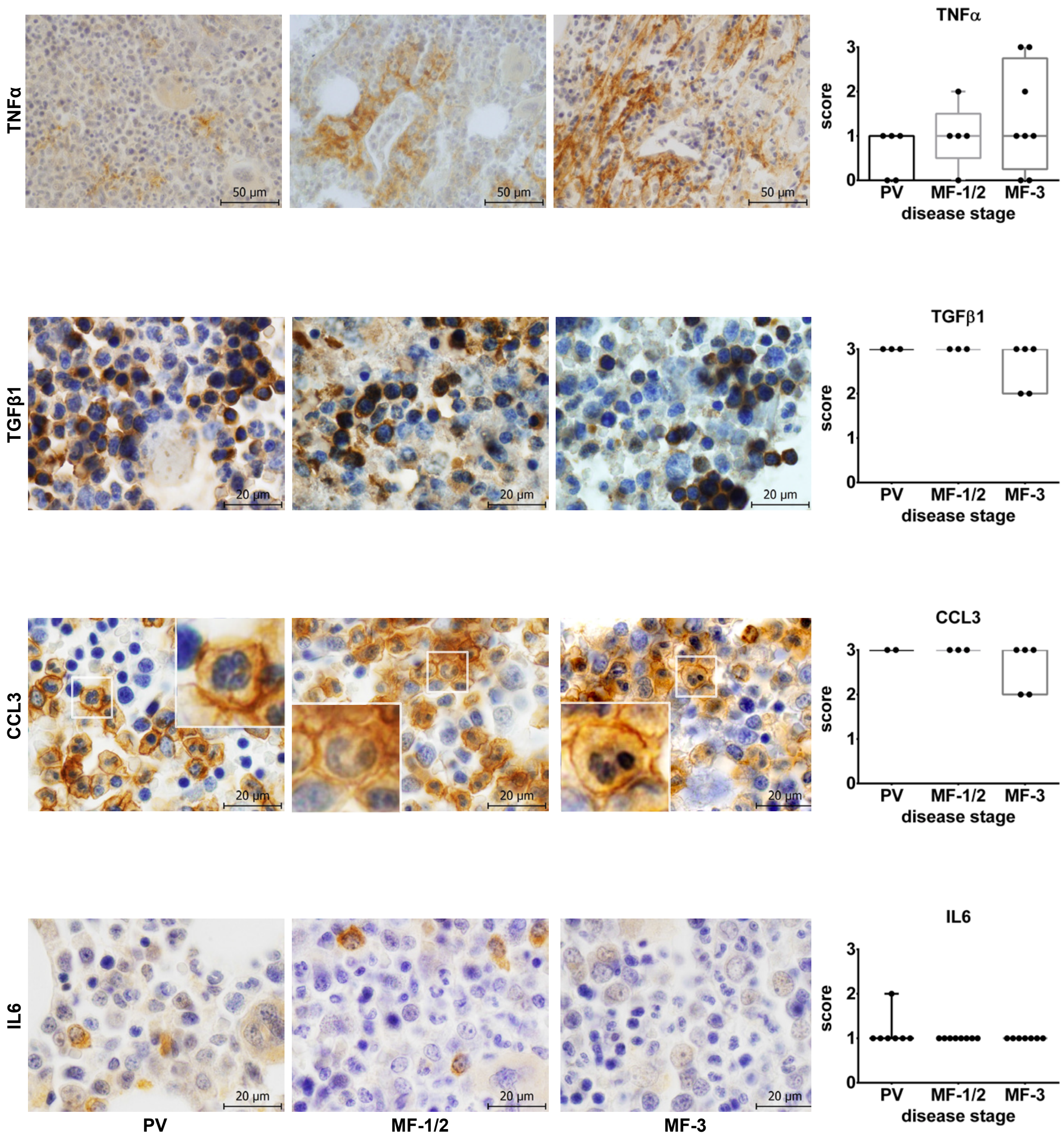

TNFα
50 µm
50 µm
50 µm
TNFα
score
3
2
1
0
PV
MF-1/2
MF-3
disease stage
TGFβ1
20 µm
20 µm
20 µm
TGFβ1
score
PV
MF-1/2
MF-3
disease stage
CCL3
20 µm
20 µm
20 µm
CCL3
score
PV
MF-1/2
MF-3
disease stage
IL6
20 µm
20 µm
20 µm
IL6
score
PV
MF-1/2
MF-3
disease stage
PV
MF-1/2
MF-3
