# Supplementary figures and images for: Addiction to DUSP1 protects JAK2V617F-driven polycythemia vera progenitors against inflammatory stress and DNA damage, allowing chronic proliferation
Source: Oncogene. 2019 Apr 9;38(28):5627–42. doi: 10.1038/s41388-019-0813-7 (PMC6756199; doi:10.1038/s41388-019-0813-7)

a

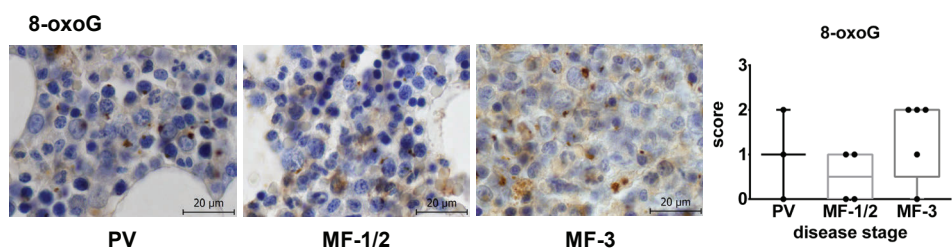

b

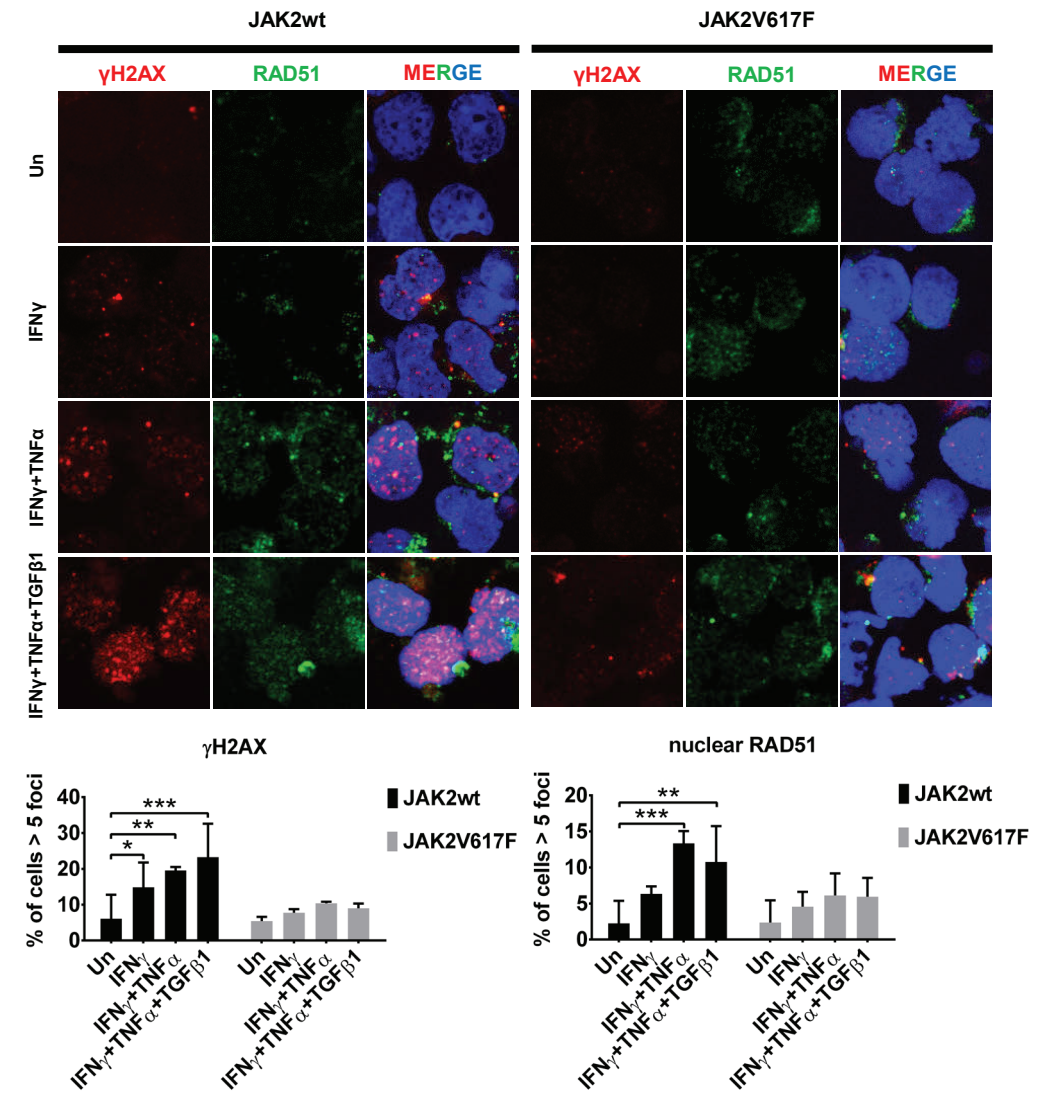

c

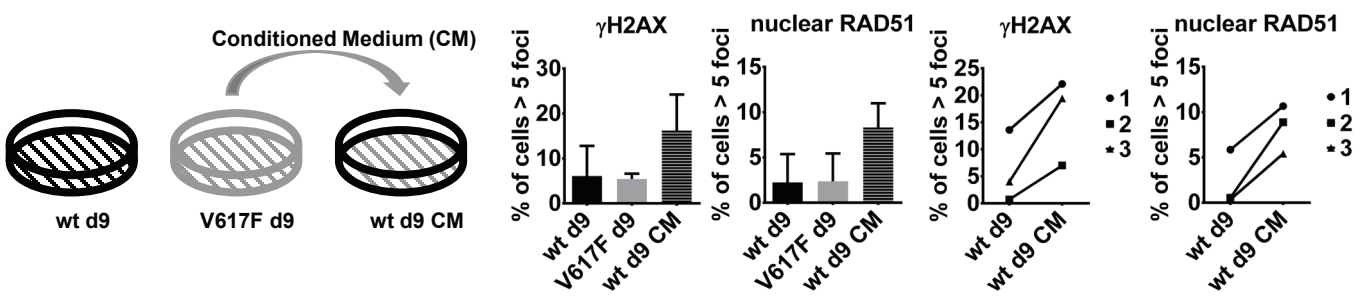

**d**

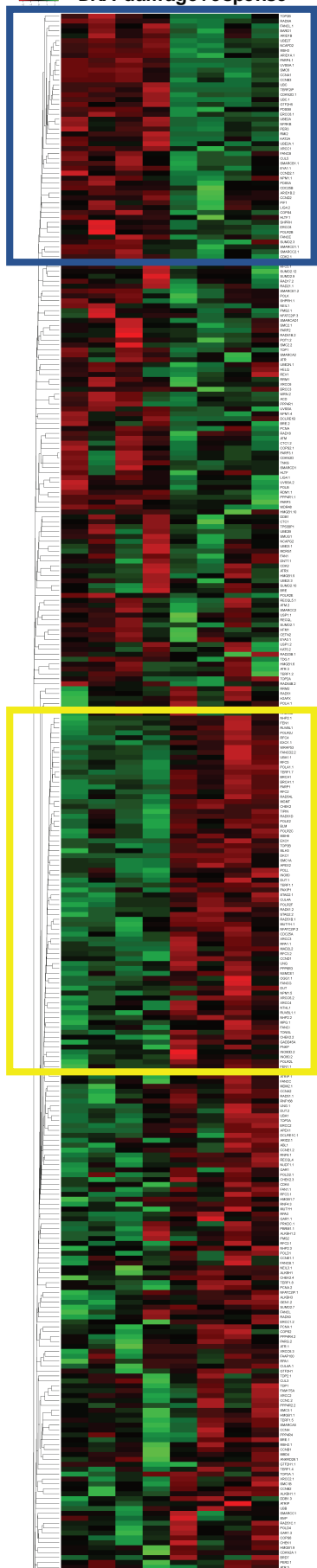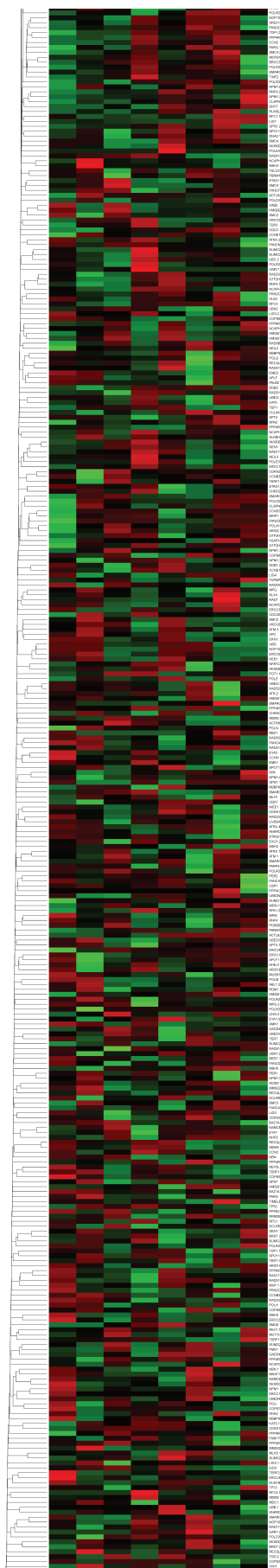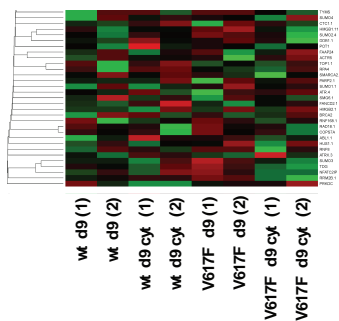

d

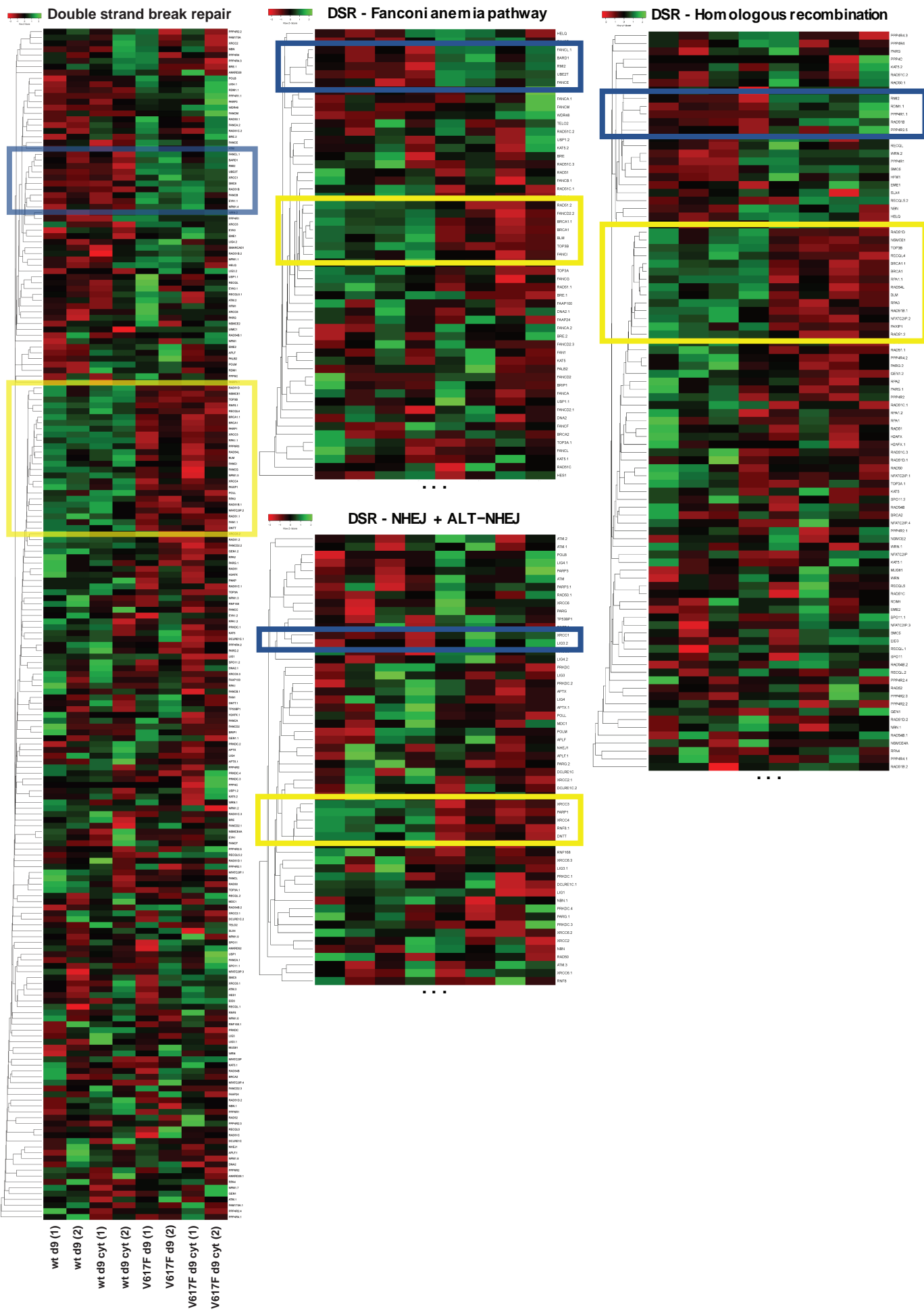

e

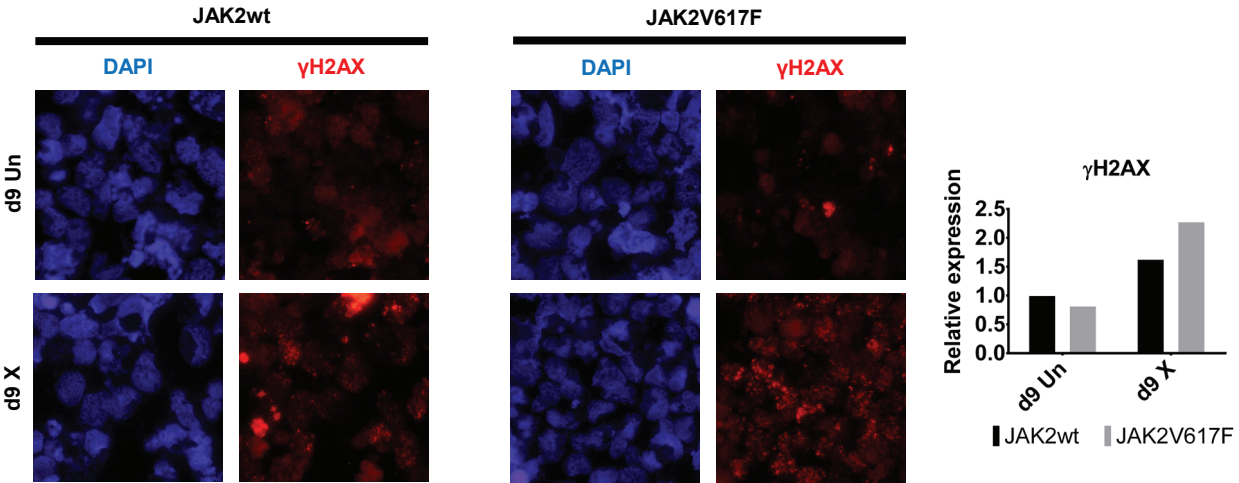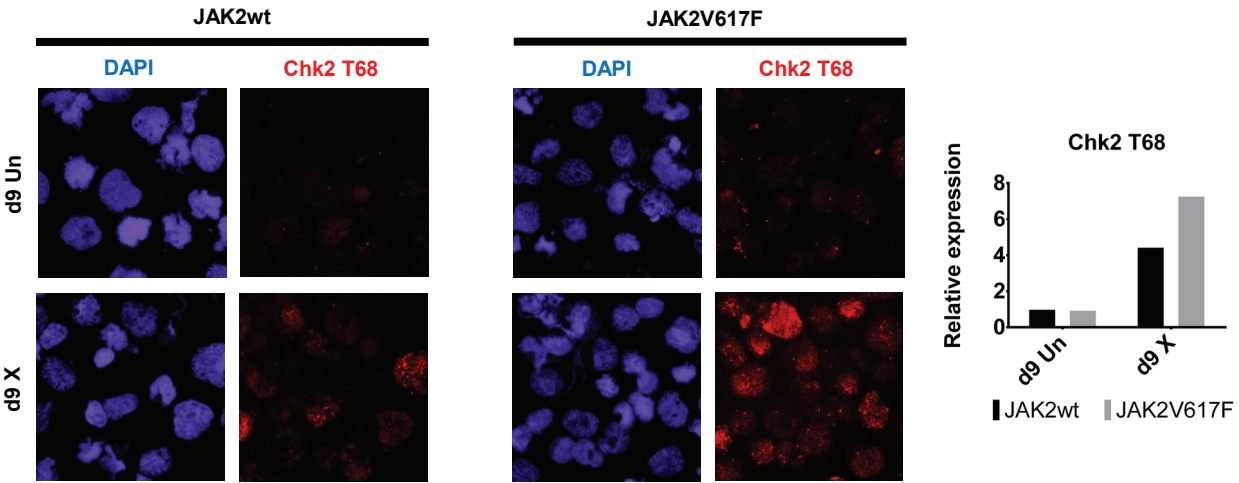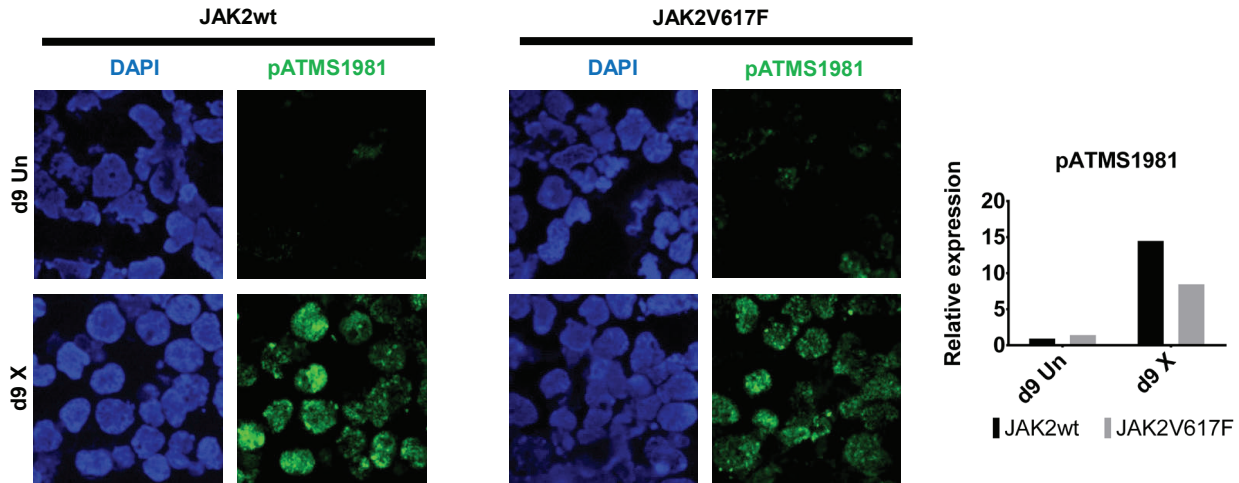

f

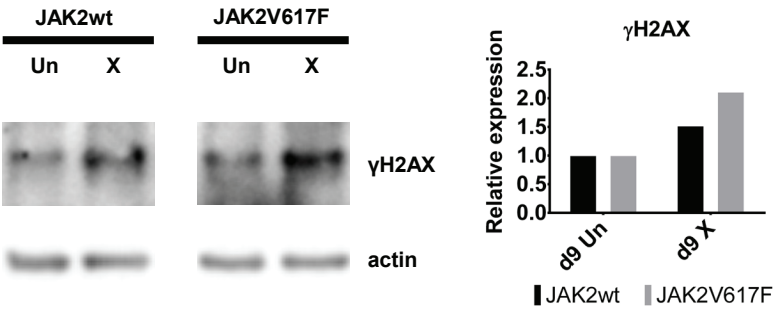

Supplement: Supplementary file 3 — Supplementary Figure 2 [file 41388_2019_813_MOESM3_ESM.pdf]

a

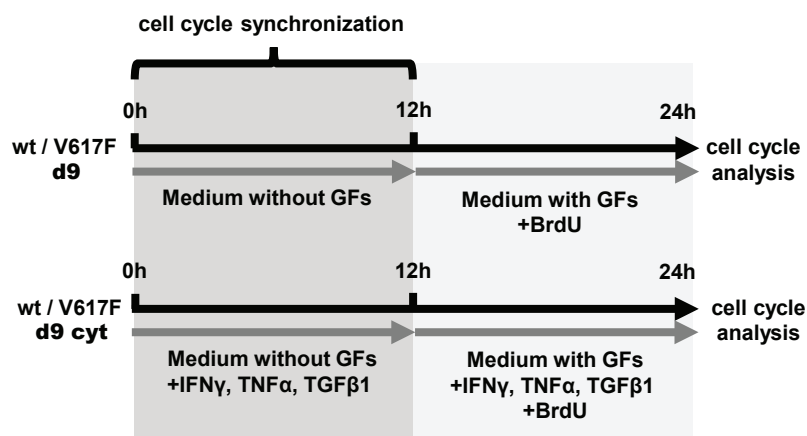

b

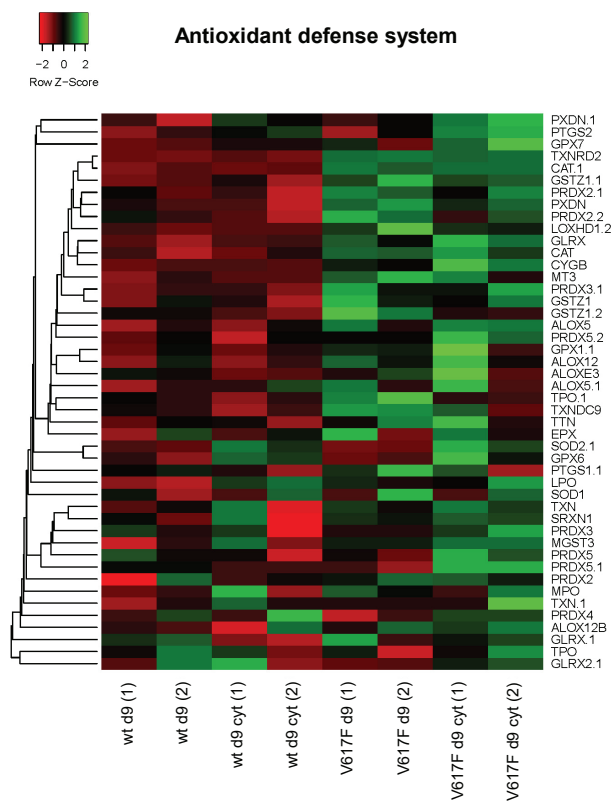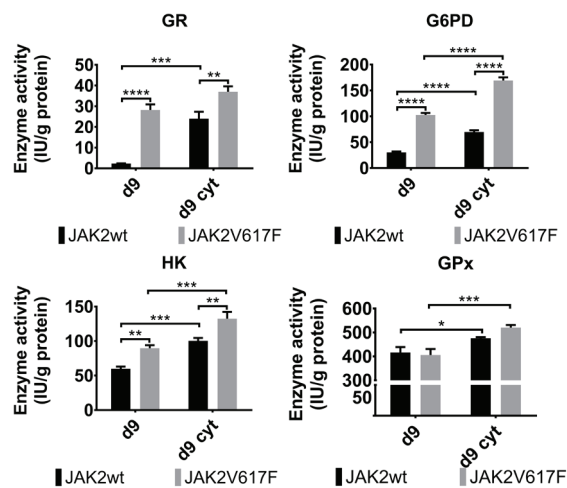

Supplement: Supplementary file 4 — Supplementary Figure 3 [file 41388_2019_813_MOESM4_ESM.pdf]

**a**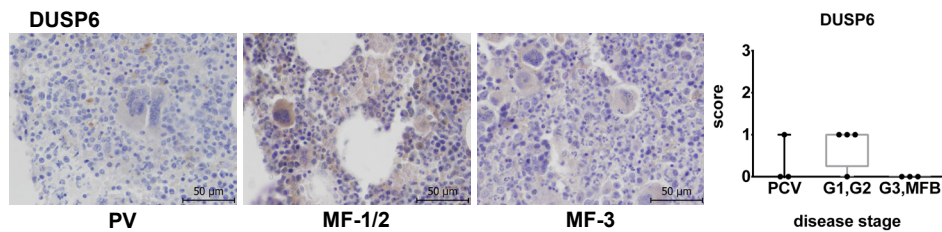**b**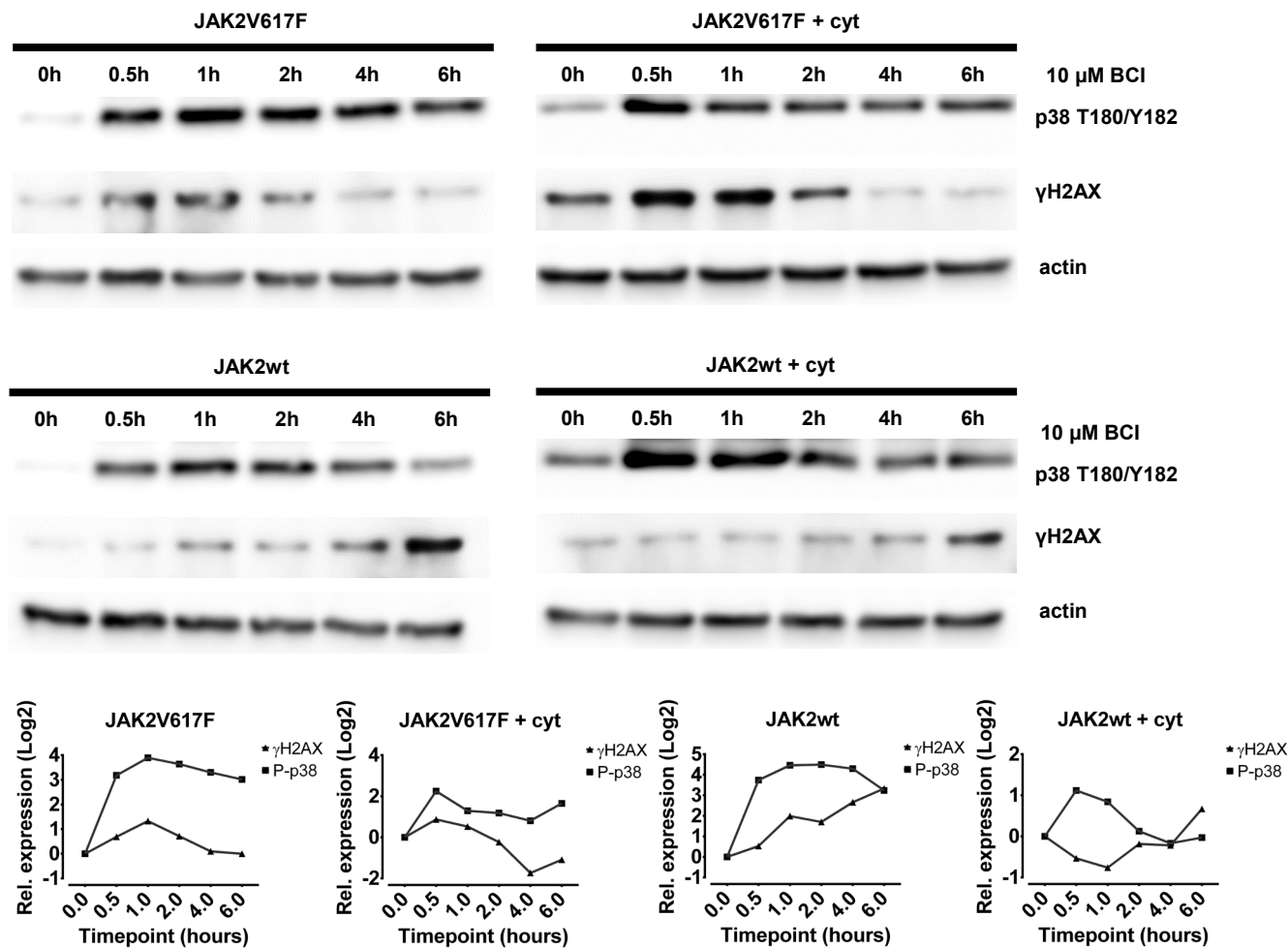**c**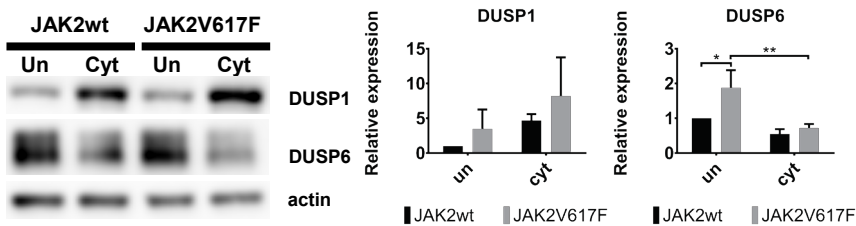**d**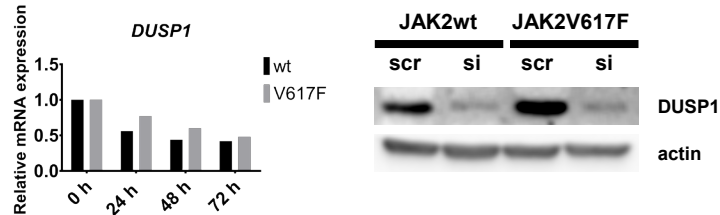**e**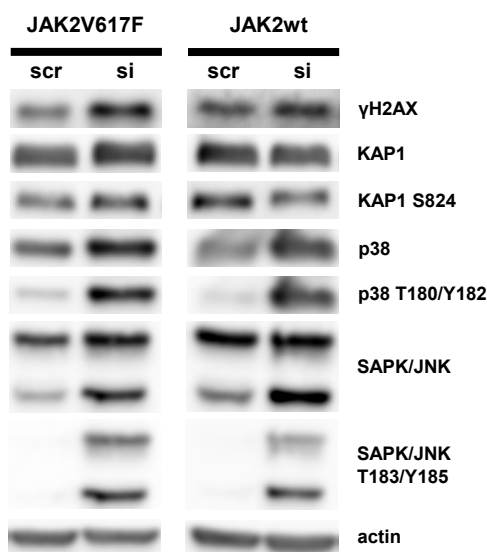

Supplement: Supplementary file 5 — Supplementary Figure 4 [file 41388_2019_813_MOESM5_ESM.pdf]
