# Supplementary material for: Addiction to DUSP1 protects JAK2V617F-driven polycythemia vera progenitors against inflammatory stress and DNA damage, allowing chronic proliferation
Source: Oncogene. 2019 Apr 9;38(28):5627–42. doi: 10.1038/s41388-019-0813-7 (PMC6756199; doi:10.1038/s41388-019-0813-7)
Supplement: Supplementary file 6 — Supplementary Materials and Methods [file 41388_2019_813_MOESM6_ESM.doc]

**SUPPLEMENTARY MATERIALS AND METHODS**

**iPSC hematopoietic differentiation**

Workflow of iPSCs differentiation is depicted below. Briefly, to generate embryoid bodies (EBs), pre-split, feeder-depleted iPSCs were treated by 1 mg/ml Collagenase B (Roche) for 10 min and 1 min by TrypLE select (Thermo Fisher). Gently scraped small colony fragments were resuspended in StemPro-34 SFM (Thermo Fisher), transferred to 6 well plate with Ultra-Low Attachment surface (Corning) and cultivated under hypoxic conditions (90% N_2_, 5% CO_2_ and 5% O_2_) at 37°C (day 0). Holo-transferrin (150 mg/ml), BMP-4 (10 ng/ml), bFGF (5 ng/ml), activin A (3 ng/ml), VEGF (15 ng/ml), DKK-1 (150 ng/ml), IL-6 (10 ng/ml), IGF-1 (25 ng/ml), IL-11 (5 ng/ml), SCF (50 ng/ml), EPO (2 U/ml final), TPO (30 ng/ml), IL-3 (30 ng/ml), and FLT3LG (10 ng/ml) were added as indicated in the schematic figure.

**
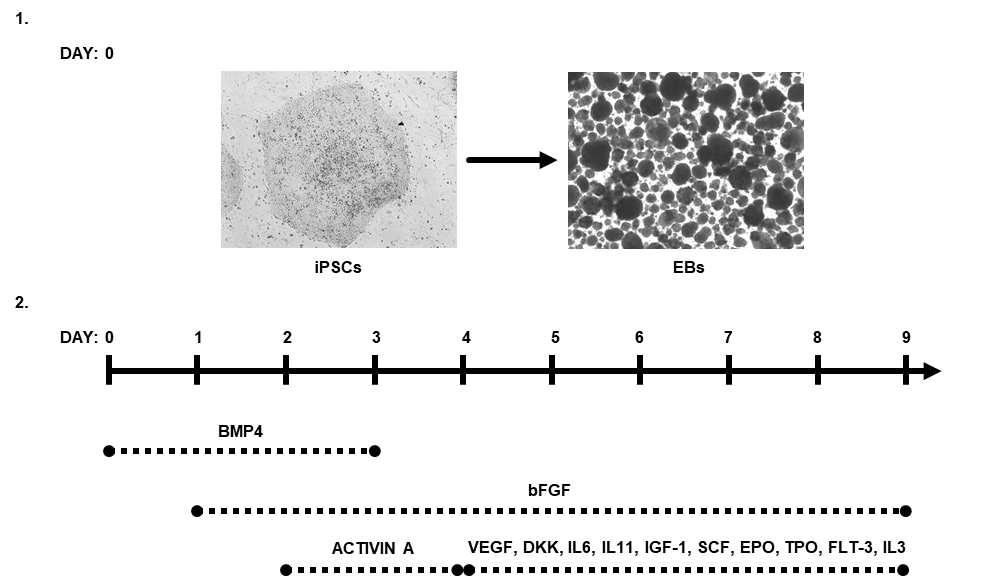
**

**Characterization of JAK2-corrected (JAK2wt) HEL cell line**

JAK2V617F mutation in HEL cells was repaired by homologous recombination using CRISPR/Cas9 system (CRISPR construct pXPR_001; Addgene #49535). A guide RNA sequence used for targeting of the *JAK2* gene was 5’-ACGAGAGTAAGTAAAACTAC-3’. A homologous template carrying *JAK2* wt sequence (1818 bp) was cloned into pGEM-T easy vector (Promega) and wobbled (protospacer adjacent motif sequence mutated to prevent CRISPR cleavage *in vitro* while preserving the amino-acid sequence). HEL cells were electroporated using Amaxa™ Nucleofector™ system (according to manufacturer’s protocol, kit V, program X-005). Cells were single-cell sorted 48 h post electroporation based on their fluorescent status and single cell clones were expanded. Due to poor survival of cells after the cell sorting this procedure was repeated several times in order to obtain specific JAK2 clones. Nevertheless, the targeted clones had only one allele edited; the second allele was either knock-out (KO) or original V617F allele (for further experiments was selected line JAK2 wt/KO, designated here as JAK2wt).

To assess the functional consequences of *JAK2* targeting in HEL JAK2wt clone on JAK/STAT signaling, we employed a dual luciferase assay using STATs responsive elements reporter plasmid (pGl4.26/pGRR4). The activity of JAK-STAT signal was significantly decreased in HEL JAK2wt clone compared to the activity of a parental control, unedited V617F^+^ clone (A). HEL JAK2wt clone exhibited statistically significant decrease of *JAK2* expression to about 50% of the control clone (B). These data were supported by immunoblotting which shows much lower levels of phosphorylated JAK2 protein in HEL JAK2wt clone. Less JAK2 protein in total was also produced in HEL JAK2wt clone compared to the control. As a result, decreased levels of phosphorylated STAT5 were detected while the total amount of STAT5 remained the same (C).


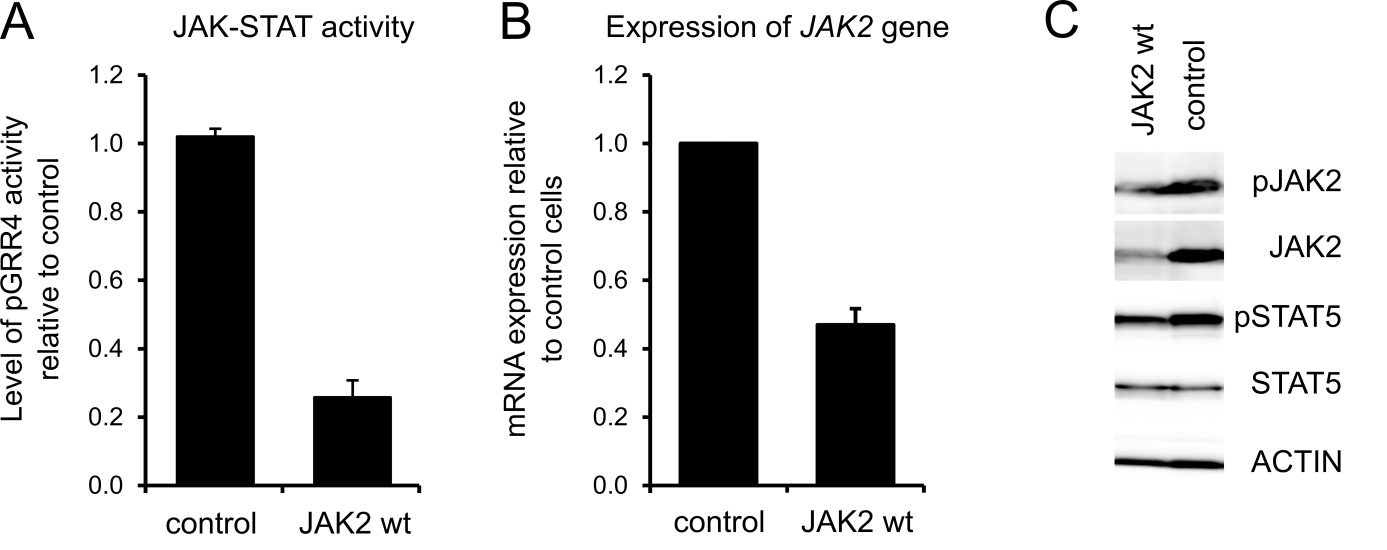


**Gating strategy used in flow cytometry of cell cycle analysis of CD34^+^ P-EC**

Gating strategy used in dual pulse labeling with PI and BrdU incorporation. All measured samples were then measured with same gating set-up.


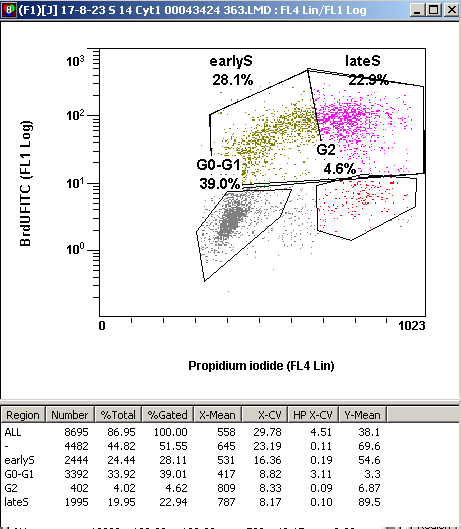


**Immunohistochemistry of patients’ samples - visualization of antigens, scoring and staining of control tissues**

Antigen unmasking was done in the microwave histoprocessor (Histos 3, Milestone, Shelton, CT) by 10 mM citrate buffer, pH 6.0, 120°C, using high pressure, for 15 min with gradual cooling. The following detection system was used: Dako EnVision+ Dual Link System-HRP (polymer labeled by HRP) and conjugated with secondary antibodies (DAKO, Glostrup, Denmark). Liquid DAB+ Substrate Chromogen System (DAKO, Glostrup, Denmark) was used for visualization of reaction. All washing steps were made twice in 0.5 M Tris-HCl, pH 7.6 (5 min each) and once in 0.5 M Tris-HCl, pH 7.6 with 0.5% Tween 20 (5 min). Finally, cell nuclei were counterstained by hematoxylin and the slide was coverslipped.

Scoring for individual antigen staining was based on 4 score values (0, 1, 2, 3) which were assigned to the percentage of positive cells stained. 0 represents negative staining, 1 corresponds to 25% of positivity, 2 to 25 - 50% positivity in the specimen and positivity over 50% is characterized by score 3. Cell positivity counts were done under the magnification × 100 and 3 fields of view/sample were observed. In the case of γH2AX staining, the scoring system was modified. The overall γH2AX positivity was weak, so following score was used: value 0 = 0 of positive cells/field of view; value 1 = 1 - 2 of positive cells/field of view and 2 = 3 and more positive cells/field of view; totally 10 fields of view per sample were evaluated. In the case of 8-oxoG, the evaluation of megakaryocytes (MKs) positivity for this factor was performed.

Selected primary antibodies were tested on positive control (PC) tissue specimens, as shown below for γH2AX, CXCL10, CXCL9, pATRT1989, 8-oxoG, TGFβ1, TNFα, pATMS1981, IFNγ, DUSP1, DUSP6, IL6 and CCL3.


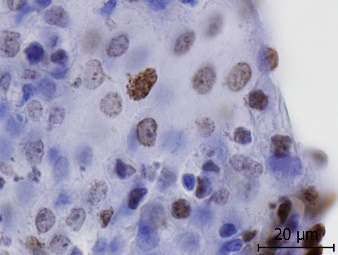


**PC - γH2AX**

**Tonsil**


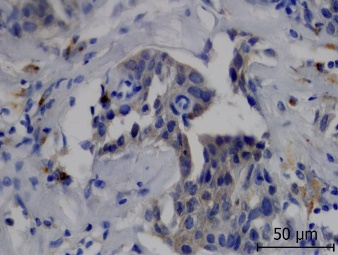


**PC – CXCL9**

**Breast carcinoma**


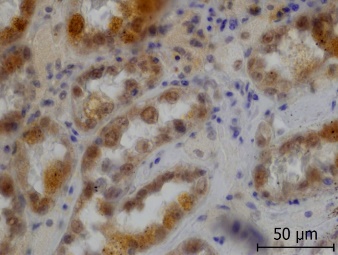


**Kidney adenocarcinoma**

**PC – CXCL10**

**PC – 8-oxoG**

**Tonsil**


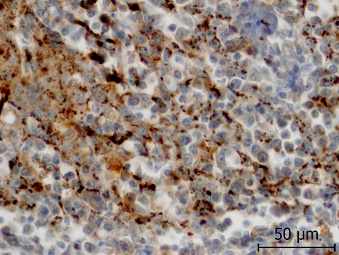


**PC – TGFβ1**

**Prostate cancer**


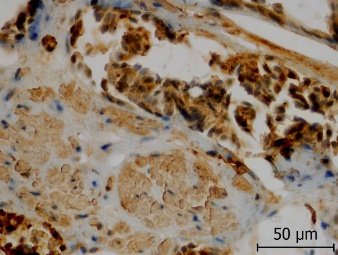

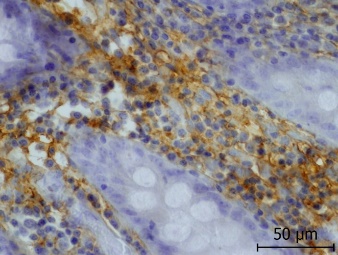


**PC – TNFα**

**Colon**


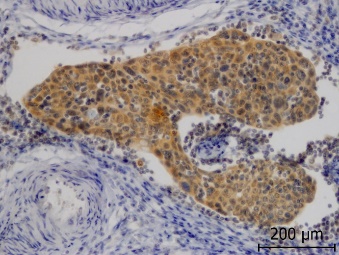


**PC – IFNγ**

**Cervical squamous**

**cell carcinoma**


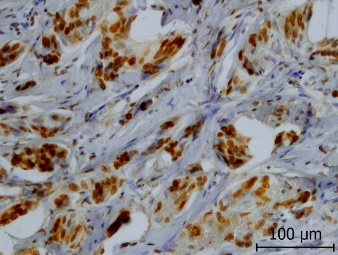


**PC – pATMS1981**

**Breast carcinoma**

**PC – pATRT1989**

**Colon**

**PC – DUSP1**

**Heart**


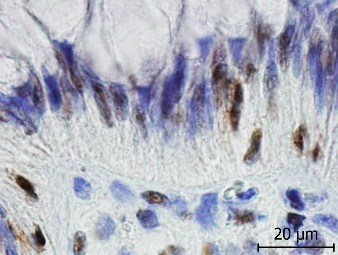

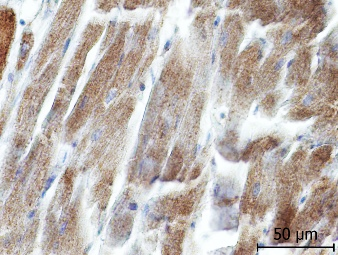

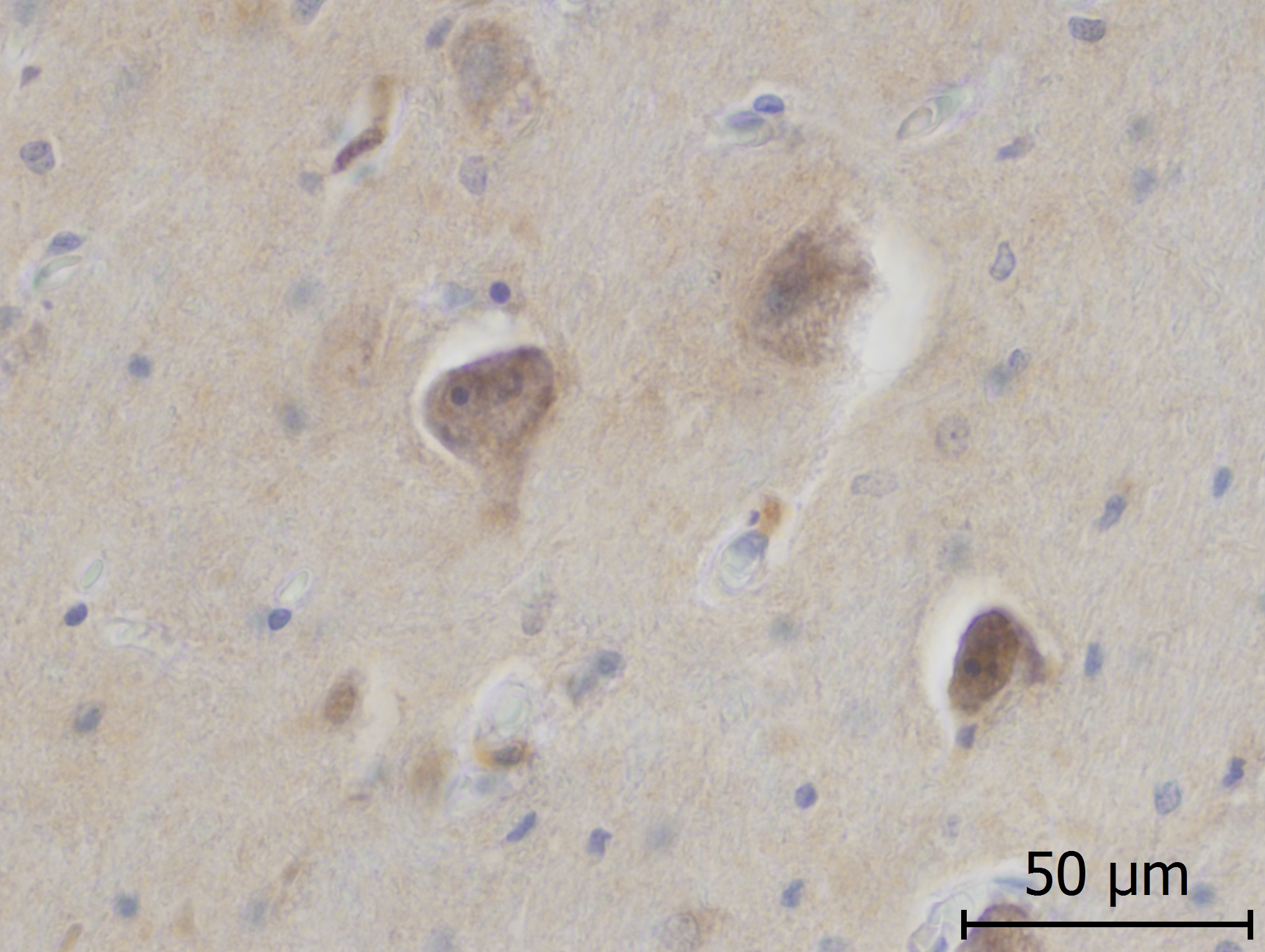


**PC – DUSP6**

**Brain**

**Islet of Langerhans**

**PC – IL6**

**PC – CCL3**

**Lung adenocarcinoma**


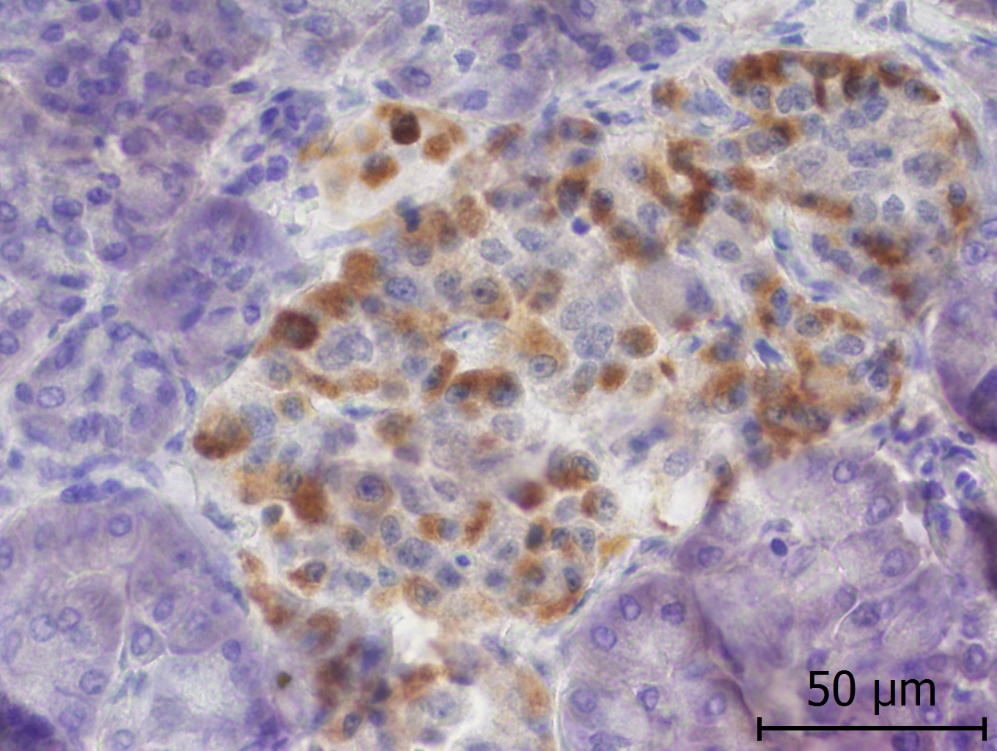

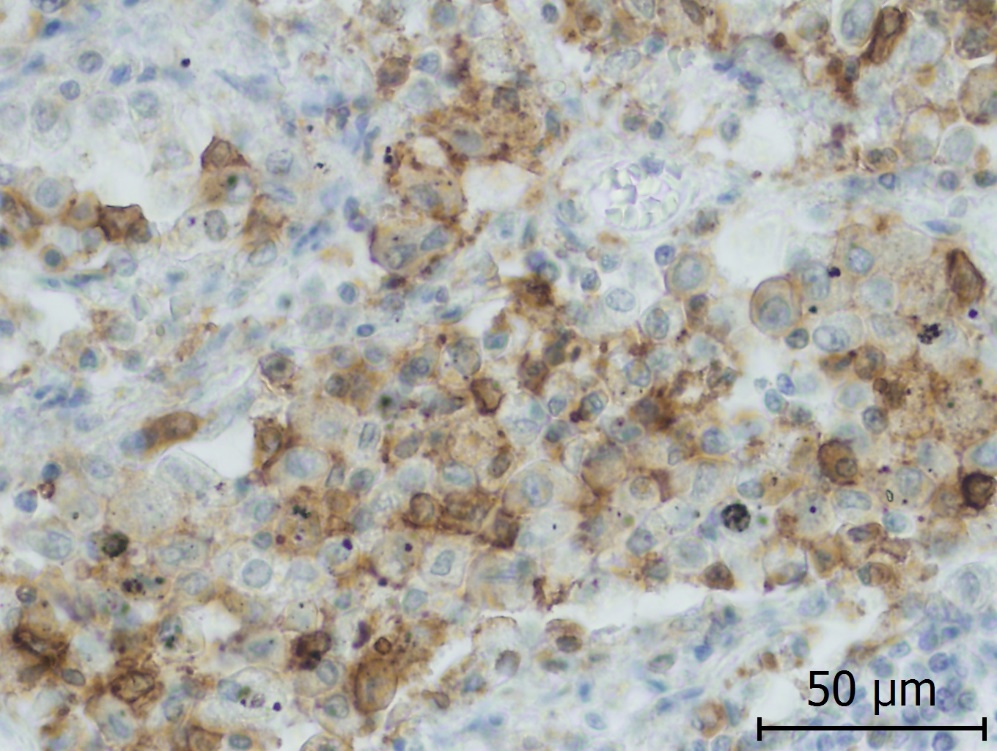


**SUPPLEMENTARY TABLES**

**Supplementary Table 1**: Primers used in the study

| **GENE NAME** | **LEFT PRIMER** | **RIGHT PRIMER** |
| --- | --- | --- |
| *RPLP0* | 5´-acagggcgacctggaagt-3´ | 5´-atctgctgcatctgcttgg-3´ |
| *CXCL10* | 5´-gaaagcagttagcaaggaaaggt-3´ | 5´-gacatatactccatgtagggaagtga-3´ |
| *CXCL9* | 5´-tgttcccctttgcttcattc-3´ | 5´-gaaaggcactgcattgtgg-3´ |
| *IFNγ* | 5´-ggcattttgaagaattggaaag-3´ | 5´-tttggatgctctggtcatctt-3´ |
| *CDKN1A* | 5´-gtacttggagtattggggtctga-3´ | 5´-cagtccaggccagtatgttacag-3´ |

**Supplementary Table 2**: Antibodies used in the study

| **ANTIGEN** | **HOST** | **CLONALITY** | **CLONE** | **DILUTION** | **PRODUCT N.** | **COMPANY** |
| --- | --- | --- | --- | --- | --- | --- |
| 8-oxoG | Mouse | mono | 2Q2311 | 1:400 | ab64548 | Abcam |
| actin | Rabbit | poly | - | 1:1000 | A-2668 | Sigma-Aldrich |
| BrdU, FITC | Mouse | mono | BMC9318 | 1:20 | 11202693001 | Roche |
| CCL3 | Rabbit | poly | - | 1:100 | ab32609 | Abcam |
| CD34 | Mouse | mono | 4H11(APG) | 1:500 | ab762 | Abcam |
| CD34, FITC | Mouse | mono | 581 | 1:20 | CD34-581-01 | Thermo Fisher |
| CD41a, PerCP-Cy5.5 | Mouse | mono | HIP8 | 1:5 | 333148 | BD Biosciences |
| CD43, APC | Mouse | mono | L10 | 1:20 | MHCD4305 | Thermo Fisher |
| CXCL10 | Rabbit | poly | - | 1:100 | ab9807 | Abcam |
| CXCL9 | Rabbit | poly | - | 1:1000 | ab9720 | Abcam |
| DUSP1 | Rabbit | poly | - | 1:50 | ab61201 | Abcam |
| DUSP1 | Mouse | mono | E-6 | 1:200 | sc-373841 | SANTA CRUZ BIOTECHNOLOGY |
| DUSP6 | Rabbit | mono | EPR129Y | 1:50 | ab76310 | Abcam |
| H2AX (pS139)/γ-H2AX | Rabbit | mono | 20E3 | 1:50 | 9718 | Cell Signaling Technology |
| H2AX (pS139)/γ-H2AX | Mouse | mono | JBW301 | 1:500 | 05-636 | Millipore |
| Chk1 | Mouse | mono | DCS-310 | 1:1000 | C9358 | Sigma-Aldrich |
| Chk1 S317 | Rabbit | poly | - | 1:500 | 2344S | Cell Signaling Technology |
| Chk2 | Mouse | mono | DCS-273 | 1:1000 | C9108 | Sigma-Aldrich |
| Chk2 Thr68 | Rabbit | poly | - | 1:500 | 2661S | Cell Signaling Technology |
| IFNγ | Rabbit | poly | - | 1:750 | ab9657 | Abcam |
| IL6 | Rabbit | Poly | - | 1:500 | ab154367 | Abcam |
| KAP1 | Rabbit | Mono | EPR5249 | 1:10 000 | ab109545 | Abcam |
| KAP1 S824 | Rabbit | poly | - | 1:500 | ab70369 | Abcam |
| p21waf1 | Rabbit | poly | C19 | 1:500 | sc-397 | SANTA CRUZ BIOTECHNOLOGY |
| p38 | Rabbit | poly | - | 1:1000 | 9212 | Cell Signaling Technology |
| p38 T180/Y182 | Rabbit | poly | - | 1:500 | 9211 | Cell Signaling Technology |
| p53 | Mouse | mono | 1C12 | 1:1000 | 2524 | Cell Signaling Technology |
| p53 S15 | Rabbit | poly | - | 1:500 | 9284S | Cell Signaling Technology |
| pATMS1981 | Mouse | mono | 7C10D8 | 1:500 | 200-301-500 | Rockland Immunochemicals |
| pATRT1989 | Rabbit | poly | - | 1:100 | ab227851 | Abcam |
| RAD51 | Rabbit | poly | - | 1:250 | ab63801 | Abcam |
| SAPK/JNK | Rabbit | poly | - | 1:1000 | 9252 | Cell Signaling Technology |
| SAPK/JNK T183/Y185 | Rabbit | poly | - | 1:500 | 9251 | Cell Signaling Technology |
| STAT1 | Rabbit | poly | - | 1:1000 | 9172 | Cell Signaling Technology |
| STAT1 Tyr701 | Rabbit | mono | 58D6 | 1:1000 | 9167 | Cell Signaling Technology |
| TGFβ1 | Rabbit | poly | - | 1:1000 | ab92486 | Abcam |
| TNFα | Rabbit | poly | - | 1:100 | ab6671 | Abcam |

**Supplementary Table 3**: Patients’ samples used in the study

|  | **PV (MF-0);**  **n = 8** | **post-PV MF-1/2; n = 14** | **post-PV MF-3;**  **n = 9** |
| --- | --- | --- | --- |
| **PRIMODIAGNOSTIC** | 7 | 10 | 1 |
| **ONGOING DISEASE** | 1 | 4 | 8 |
| **THERAPY (hydroxyurea)** | 1 | 3 | 5; 1 unknown |
